# Dietary Improvement during Lactation Normalizes miR-26a, miR-222 and miR-484 Levels in the Mammary Gland, but Not in Milk, of Diet-Induced Obese Rats

**DOI:** 10.3390/biomedicines10061292

**Published:** 2022-05-31

**Authors:** Catalina A. Pomar, Pedro Castillo, Andreu Palou, Mariona Palou, Catalina Picó

**Affiliations:** 1 Laboratory of Molecular Biology, Nutrition and Biotechnology, Group of Nutrigenomics, Biomarkers and Risk Evaluation, University of the Balearic Islands, 07122 Palma, Spain; c.pomar@uib.es (C.A.P.); pedro.castillo@uib.es (P.C.); andreu.palou@uib.es (A.P.); cati.pico@uib.es (C.P.); 2 Health Research Institute of the Balearic Islands (IdISBa), 07010 Palma, Spain; 3 CIBER de Fisiopatología de la Obesidad y Nutrición, Instituto de Salud Carlos III, 28029 Madrid, Spain

**Keywords:** miRNA, mammary gland, lactation, obesogenic diet

## Abstract

We aimed to evaluate in rats whether the levels of specific miRNA are altered in the mammary gland (MG) and milk of diet-induced obese dams, and whether improving maternal nutrition during lactation attenuates such alterations. Dams fed with a standard diet (SD) (control group), with a Western diet (WD) prior to and during gestation and lactation (WD group), or with WD prior to and during gestation but moved to SD during lactation (Rev group) were followed. The WD group showed higher miR-26a, miR-222 and miR-484 levels than the controls in the MG, but the miRNA profile in Rev animals was not different from those of the controls. The WD group also displayed higher miR-125a levels than the Rev group. Dams of the WD group, but not the Rev group, displayed lower mRNA expression levels of *Rb1* (miR-26a’s target) and *Elovl6* (miR-125a’s target) than the controls in the MG. The WD group also presented lower expression of *Insig1* (miR-26a’s target) and *Cxcr4* (miR-222’s target) than the Rev group. However, both WD and Rev animals displayed lower expression of *Vegfa* (miR-484’s target) than the controls. WD animals also showed greater miR-26a, miR-125a and miR-222 levels in the milk than the controls, but no differences were found between the WD and Rev groups. Thus, implementation of a healthy diet during lactation normalizes the expression levels of specific miRNAs and some target genes in the MG of diet-induced obese dams but not in milk.

## 1. Introduction

Breastfeeding in mammals has evolved over millions of years to provide more than what is traditionally considered nutrition. In fact, breast milk contains not only macronutrients, minerals and vitamins, which can provide the energy and factors necessary for growth and development, but it also provides a large variety of bioactive compounds [1]. Such compounds mainly include immune factors, hormones and non-coding small RNAs—such as microRNAs (miRNAs)—that can regulate cellular signaling pathways, affect organ development and protect against infection and inflammation, among other functions, with subsequent health outcomes [2,3,4]. Regarding miRNAs, evidence suggests that breast milk is a rich source of these compounds [5], which are produced and secreted into milk primarily through endogenous synthesis in the mammary epithelium, with minor contributions from the maternal blood circulation [6]. Interestingly, specific miRNAs in milk are modulated by maternal diet and maternal weight [7,8,9] and, once consumed, they may exert an effect on the target mRNAs in specific tissues of the descendants [3,10,11]. For example, there is indirect evidence that specific miRNAs in human breast milk affected by maternal overweight/obesity may influence infants’ body mass index [8]. Moreover, certain miRNAs that are differentially expressed in breast milk from mothers with Type 1 diabetes, compared with healthy mothers, are associated with immunomodulatory effects [12], so that such alterations may affect the development of the immune system in the infants. Furthermore, alterations in the miRNA profile associated with maternal diet and/or status might also be expected to have autocrine or paracrine effects on maternal mammary function itself.

Previous studies have pointed out the importance of miRNA expression dynamics in mammary gland (MG) development and lactation [13,14]. In this sense, it has been described that the development of the MG can be affected by maternal diet. Lactating animals fed with an obesogenic diet showed an impaired lactogenesis and abnormal mammary morphology, leading to an inflammatory process [15,16]. However, the specific mechanisms involved are unclear. We have previously described how rats fed on an obesogenic diet during lactation displayed lower miR-26a levels in milk compared with dams fed a standard diet, which was in accordance with the lower expression of this specific miRNA in the MG at the end of lactation [17,18]. Therefore, alterations in the miRNA profile in the MG may be detected in the milk and reflect maternal diet and/or maternal physiological state. Thus, considering the potential impact of miRNAs on infant development, the study of how maternal nutrition and/or obesity states could affect miRNA expression in the MG and, consequently, the miRNA profile in milk, and whether such alterations may be prevented, is of great interest in the field of infant nutrition.

We have previously shown that maternal intake of an obesogenic diet during lactation in diet-induced obese rats, rather than maternal excess of adiposity (in the absence of dietary alterations during lactation), appears to be the main contributor to alterations in the macronutrient content and the levels of metabolic hormones in milk [19]. Interestingly, we have also shown that dietary normalization during lactation in diet-induced obese dams attenuates the early detrimental programming effects in the offspring [19]. Therefore, in the present study, we have analyzed a set of miRNAs (miR-26a, miR-27a, miR-30d, miR-99b, miR-125a, miR-181a, miR-200a, miR-200b, miR-222, miR-320a, miR-331 and miR-484), which have been described as being dysregulated in certain metabolic disorders [20,21,22,23,24,25,26,27,28,29], in the MG and milk of the aforementioned animal models. These consisted of diet-induced obese dams that continued on an obesogenic diet during lactation or had their diet improved during this period. We hypothesized that normalizing the diet during lactation in obese rats could potentially prevent changes in MG function and restore the miRNA profile in milk caused by the consumption of an obesogenic diet during these critical periods: gestation and lactation.

## 2. Materials and Methods

### 2.1. Study Design and Sample Collection

The study was performed on samples from a previously published study [19]. The animal protocol was approved by the Bioethical Committee of the University of the Balearic Islands (Exp. 2018/13/AEXP, 23 January 2019). Virgin female Wistar rats, housed at a controlled temperature (22 °C), with a 12-h light–dark period and with free access to food and water, were fed with a standard chow diet (SD; 3.3 kcal·g^−1^, with 8.4% calories from fat, 72.4% from carbohydrates and 19.3% from protein; Safe, Augy, France) (control group), or a high-fat and high-sucrose diet (Western diet, WD; 4.7 kcal·g^−1^, with 40.0% calories from fat, 43.0% from carbohydrates and 17.0% from proteins; Research Diets, New Brunswick, NJ, USA) for one month prior to being bred with male rats (this period was referred as pre-gestation). Pregnant dams were housed individually and continued with their assigned diets during the gestation period. On postnatal Day 1, the litters were equated to 10 pups per dam, 5 males and 5 females when possible. Throughout lactation, the dams of the control group continued with the SD (*n* = 8), but those fed with the WD either continued with the WD diet (Western diet group: WD group, *n* = 9) or were exposed to the SD during this period (reversion group: Rev group; *n* = 10). At three time points of lactation (Days 5, 10 and 15), milk samples from the dams were collected. For milk extraction, dams were separated from their pups for 2–3 h, then, 4 IU kg^−1^ of body weight of oxytocin (Facilpart, Laboratory Syva S.A.U, León, España) was administered intraperitoneally, and the dams were anesthetized using isoflurane (IsoFlo, Abbott Laboratories Ltd., North Chicago, IL, USA) throughout the extraction procedure to reduce their stress level. Milk was extracted manually from all teats and stored at −80 °C until further analysis.

The body weight of dams was followed, and body fat content was measured at the different time points by EchoMRI-700^TM^ (Echo Medical Systems, LLC, Houston, TX, USA). At weaning (Day 21), the dams were killed by decapitation during the first 2 h at the beginning of the light cycle. Maternal MG was rapidly removed and frozen in liquid nitrogen and stored at −80 °C until analysis.

### 2.2. miRNA Expression Profiling by Real-Time qPCR in the Mammary Gland

Total RNA was extracted from MGs at weaning (Day 21) using Tripure Reagent (Roche Diagnostic Gmbh, Mannheim, Germany) according to the manufacturer’s instructions. Isolated RNA was quantified using a NanoDrop ND-1000 spectrophotometer (NadroDrop Technologies, Wilmington, DE, USA). Its integrity was confirmed using agarose gel electrophoresis.

Real-time polymerase chain reaction was used to measure the miRNA expression levels in the MG. Total RNA (0.10 μg) was reverse-transcribed using the miRCURY LNA^TM^ RT Kit (Qiagen, Barcelona, Spain) at 42 °C for 60 min and 5 min at 95 °C in an Applied Biosystems 2720 Thermal Cycler (Applied Biosystem, Madrid, Spain). Real-time PCR was performed using the Applied Biosystems StepOnePlus™ Real-Time PCR system (Applied Biosystems) with the following profile: 2 min at 95 °C, followed by a total of 40 two-temperature cycles (10 s at 95 °C and 1 min at 56 °C). Each PCR was performed with 1/30 diluted cDNA using the miRCURY LNA SYBR Green PCR kit and individual miRCURY LNA miRNA PCR assayw (Qiagen) for miR-26a-5p (YP00206023), miR-27a-3p (YP00206038), miR-30d-3p (YP00204023), miR-99b-5p (YP00205983), miR-125a-5p (YP00204339), miR-181a-5p (YP00206081), miR-200a-5p (YP02102685), miR-200b-5p (YP00204144), miR-222-3p (YP00204551), miR-320a (YP00206042), miR-331-3p (YP00206046) and miR-484 (YP00205636). To verify the purity of the products, a melting curve was produced after each run according to the manufacturer’s instructions. The threshold cycle (Ct) was calculated by the instrument’s software (StepOne Software v2.3., Applied Biosystems, Madrid, Spain) and the relative expression of each miRNA was calculated by the 2^−ΔΔCt^ method using miR-191-5p (ZP00000368) as the reference miRNA [30].

### 2.3. mRNA Expression Profiling by Real-Time qPCR in the Mammary Gland

For analysis of selected mRNA of the validated target genes, 0.25 μg of total RNA was denatured at 65 °C for 10 min and then reverse-transcribed to cDNA using MuLV reverse transcriptase (Applied Biosystems) at 20 °C for 15 min and 42 °C for 30 min, with a final step of 5 min at 95 °C in an Applied Biosystems 2720 Thermal Cycler (Applied Biosystems). Each PCR was performed with diluted cDNA template, forward and reverse primers (5 μM each), and the Power SYBER Green PCR Master Mix (Applied Biosystems). Real-time PCR was performed using the Applied Biosystems StepOnePlus™ Real-Time PCR system (Applied Biosystems) with the following profile: 10 min at 95 °C, followed by a total of 40 two-temperature cycles (15 s at 95 °C and 1 min at 60 °C). To verify the purity of the products, a melting curve was produced after each run according to the manufacturer’s instructions. The threshold cycle (Ct) was calculated by the instrument’s software (StepOne Software v2.3., Applied Biosystem) and the relative expression of each mRNA was calculated by the 2^−Δ^^ΔCt^ method using Guanosine diphosphate dissociation inhibitor (*Gdi*) as the reference gene. All primers were obtained from Sigma (Sigma Aldrich Co., LLC, Madrid, Spain), and the sequences and amplicon sizes are detailed in Supplementary Table S1.

### 2.4. miRNA Target Prediction and Pathway Analyses

In silico target prediction analysis for the miRNA of interest was carried out using TargetScan [31]. Next, the altered WikiPathways were assessed with the GlueGo+CluePedia Cytoscape plugin [32,33]. Only altered pathways with a *p*-value < 0.0005 after Bonferroni correction were considered.

### 2.5. miRNA Expression Profiling by Real-Time qPCR in Milk

The levels of selected miRNA in whole milk on Days 5, 10 and 15 were analyzed. Total RNA was extracted and purified from 50 µL of milk using a mirVana miRNA Isolation kit (Life Technologies Corporation, Waltham, MA, USA) according to the manufacturer’s protocol. All samples were eluted from columns in 50 µL of RNase-free water. The quantity of the RNA was assessed using a NanoDrop ND-1000 spectrophotometer (NanoDrop Technologies Inc., Wilmington, DE, USA). The miRNA Real-Time qPCR was performed as described above using 10 ng of total RNA.

### 2.6. Statistical Analysis

Data are expressed as the mean ± SEM (*n* = 8–12). Data were checked for normality using the Shapiro–Wilks normality test, and Levene’s test was performed to assess the homogeneity of the variance between groups. Logarithmic transformation was applied when required before analysis. Differences among groups were assessed by one-way ANOVA followed by least significant difference (LSD) post-hoc comparisons. For miRNAs measured at different time points, repeated-measures ANOVA followed by LSD post-hoc tests were used to compare the mean differences among groups and/or between the time points of the lactation period. Comparisons between two groups were assessed by the non-parametric Mann–Whitney U-test. In addition, potential relationships between two variables were assessed using Pearson’s correlation coefficient. The test used for each comparison is indicated in the footnotes of the figures. The threshold of significance was defined at *p* < 0.05. The analyses were performed with SPSS for Windows (SPSS, Chicago, IL, USA).

## 3. Results

### 3.1. Summary of Previously Described Phenotypic Traits of Dams

The previously published results of the phenotypic traits of dams are summarized in Table 1 [19]. Before mating, after being exposed to an obesogenic diet for one month, rats (WD and Rev groups) displayed a greater body fat content compared with the control rats (one-way ANOVA, post-hoc analysis). Interestingly, at the end of lactation, the dams of the WD group displayed a lower body weight than the control and Rev groups, and a lower fat content (g) than Rev animals (one-way ANOVA, post-hoc analysis), whereas dams of the Rev group maintained excess body fat compared with the controls (one-way ANOVA, post-hoc analysis). Thus, as previously described [19], the negative energy balance characteristic of the lactation period for coping with milk production seems to be more marked in dams of the WD group. Therefore, the study of obese dams exposed to a SD during lactation (Rev group), together with obese dams that continued with the obesogenic diet during this period (WD group), is of interest to evaluate whether dietary improvements during lactation could attenuate possible alterations in the specific miRNA levels associated with maternal exposure to an obesogenic diet, both in the MG (at weaning, Day 21 of lactation) and in milk (Days 5, 10 and 15 of lactation).

### 3.2. miRNA Profile in the Mammary Gland

**Figure 1:**
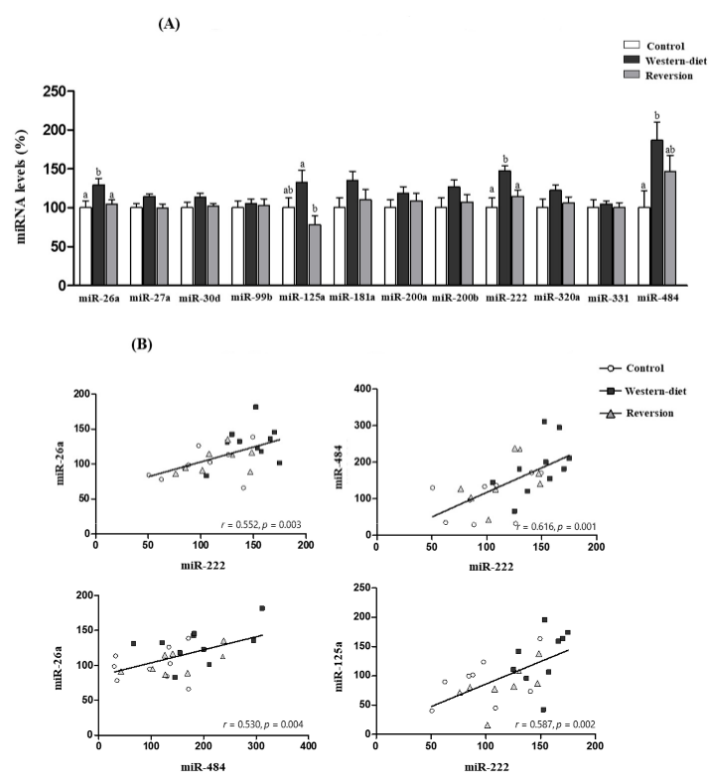
(**A**) miRNA levels in the mammary gland on Day 21 of lactation in control, Western diet and reversion animals. (**B**) Correlations between altered miRNAs in the mammary gland. The selected miRNA levels (miR-26a, miR-27a, miR-30d, miR-99b, miR-125a, miR-181a, miR-200a, miR-200b, miR-222, miR-320a, miR-331 and miR-484) were measured by RT-qPCR. Values are presented as percentages versus the levels of the controls. Data are expressed as the mean ± SEM of 8–10 animals per group. Statistics: Least significant difference (LSD) post-hoc test, a ≠ b (effect of maternal diet, one-way ANOVA). The correlations were assessed by Pearson’s correlation (2-tailed). Pearson’s correlation coefficients (r) and *p*-values are indicated.

The levels of selected miRNAs (miR-26a, miR-27a, miR-30d, miR-99b, miR-125a, miR-181a, miR-200a, miR-200b, miR-320a, miR-331 and miR-484) in the MG of dams at weaning were analyzed (Figure 1A). Dams of the WD group displayed higher expression levels of miR-26a and miR-222 in MG compared with the control (29% and 47%, respectively) and Rev (23% and 28%, respectively) groups (one-way ANOVA, post-hoc analysis). miR-484 expression levels in the MG of WD animals were increased compared with the controls (87%), whereas the expression levels in Rev rats were not different from those in the control and WD groups (one-way ANOVA, post-hoc analysis). In addition, the WD group displayed greater miR-125a expression in MG compared with the Rev group (69%) but not compared with the controls (one-way ANOVA, post-hoc analysis). Correlation analyses between differentially expressed miRNAs in the MG that were significant (Pearson’s correlation) are shown in Figure 1B. miR-26a, miR-222 and miR-484 levels were positively correlated with each other (miR-222 and miR-26a (r = 0.552, *p* = 0.003), miR-222 and miR-484 (r = 0.616, *p* = 0.001), miR-484 and miR-26a (r = 0.530, *p* = 0.004), and miR-125a levels correlated positively with miR-222 levels (r = 0.587, *p* = 0.002).

### 3.3. Expression of Validated Target Genes in the Mammary Gland

**Figure 2:**
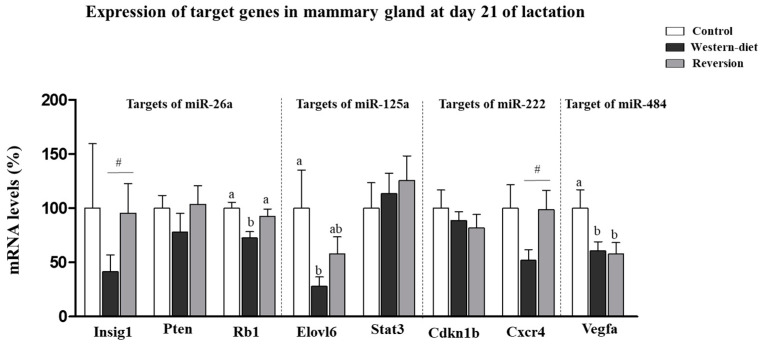
Expression of target genes of altered miRNAs (miR-26a, miR-125a, miR-222 and miR-484) in the mammary gland on Day 21 of lactation in control, Western diet and reversion dams. mRNA levels were measured by RT-qPCR. The genes determined were insulin induced gene 1 (*Insig1*), phosphatase and tensin homolog (*Pten*), RB transcriptional corepressor 1 (*Rb1*), ELOVL fatty acid elongase 6 (*Elovl6*), signal transducer and activator of transcription 3 (*Stat3*), cyclin-dependent kinase inhibitor 1B (*Cdkn1b*), C-X-C motif chemokine receptor 4 (*Cxcr4*) and vascular endothelial growth factor A (*Vegfa*). Values are presented as percentages versus the levels of the controls. Data are expressed as the mean ± SEM of 8–10 animals per group. Statistics: Least significant difference (LSD) post-hoc test, a ≠ b (effect of maternal diet, one-way ANOVA). #, Western diet versus reversion dams (*p* < 0.05, Mann–Whitney U-test).

Next, we examined whether the expression of a subset of target genes for the altered miRNAs was affected in the MG. Specifically, the gene expression of insulin induced gene 1 (*Insig1*), phosphatase and tensin homolog (*Pten*) and RB transcriptional corepressor 1 (*Rb1*) (targets of miR-26a [17,34]); ELOVL fatty acid elongase 6 (*Elovl6*) and signal transducer and activator of transcription 3 (*Stat3*) (targets of miR-125a [35,36]); cyclin-dependent kinase inhibitor 1B (*Cdkn1b*) and C-X-C motif chemokine receptor 4 *(Cxcr4)* (targets of miR-222 [37,38]) and vascular endothelial growth factor A (*Vegfa*) (a target of miR-484 [39]) were selected, as they have been previously validated as target genes (Figure 2). The WD group but not the Rev group displayed lower mRNA expression levels of *Rb1* (27.1%) and *Elovl6* (71.9%) than the controls (one-way ANOVA, post-hoc analysis). In addition, WD animals displayed lower mRNA expression levels of *Insig1* (57%) and *Cxcr4* (47%) than the Rev group (Mann–Whitney U test). Interestingly, both WD and Rev animals displayed lower mRNA expression levels of *Vegfa* than the controls (39% and 42%, respectively) (one-way ANOVA, post-hoc analysis).

### 3.4. In Silico Prediction of Altered Pathways in the Mammary Gland

To gain insight into the potential role of those miRNAs (miR-26a, miR-222 and miR-484), whose levels were altered in the MG of dams of the WD group but not of the Rev group, and to ascertain in which pathways could be involved, putative target genes were searched with the online algorithm for miRNA target prediction, TargetScan. The resulting list of 1699 predicted target genes recognized by ClueGO were used to assess the altered WikiPathways (Supplementary Table S2). The pathway analysis identified eight significantly altered pathways with *p*-values of <0.0005 (Table 2), namely the VEGFA-VEGFR2 signaling pathway (WP:3888), endoderm differentiation (WP:2853), the brain-derived neurotrophic factor (BDNF) signaling pathway (WP:2380), insulin signaling (WP:481), the mesodermal commitment pathway (WP:2857), ErbB signaling (WP:673), melanoma (WP:4685) and hematopoietic stem cell gene regulation by the GABP alpha/beta complex (WP:3657). It is remarkable that 74 target genes were related to the VEGFA-VEGFR2 signaling pathway (16.9% of the associated genes), 38 were related to endoderm differentiation (26.0% of the associated genes), 36 were related to the brain-derived neurotrophic factor (BDNF) signaling pathway (25.0 % of the associated genes) and 36 were related to the insulin signaling pathway (22.4% of the associated genes). These results suggest the potential dysregulation of the abovementioned pathways due to miRNA dysregulation in WD animals, but not in Rev, compared with the controls.

### 3.5. miRNA in Milk at Different Time Points of Lactation

**Figure 3:**
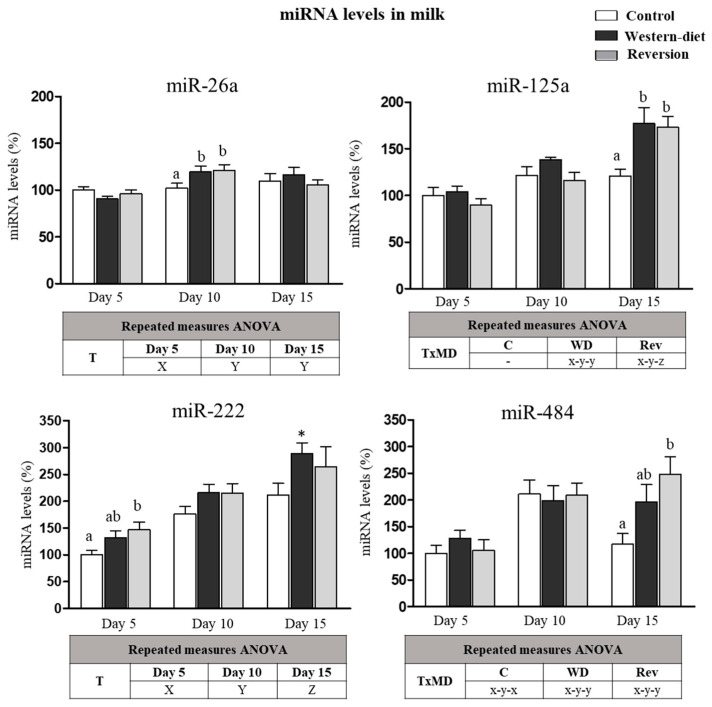
miRNA levels in milk on Days 5, 10 and 15 of lactation in the control, Western diet and reversion groups. Milk samples were collected at different time points of lactation (Days 5, 10 and 15). The selected miRNA levels (miR-26a, miR-125a, miR-222 and miR-484) were measured by RT-qPCR. Values are presented as percentages versus the levels of the controls on Day 5. Data are expressed as the mean ± SEM of 8–10 animals per group. Statistics: T, effect of time; TxMD, interactive effect between time and maternal diet (repeated-measures ANOVA). Least significant difference (LSD)post-hoc test, X ≠ Y ≠ Z (effect of time, repeated-measures ANOVA), x ≠ y ≠ z (effect of time, one-way ANOVA) and a ≠ b (effect of maternal diet, one-way ANOVA). *, Western diet versus control dams (*p* < 0.05, Mann–Whitney U-test).

In view of the differences among the groups found in the levels of certain miRNAs in the MG and considering that miRNA from the MG can be released into the milk, the levels of the altered miRNAs were analyzed in milk at three time points of lactation (Days 5, 10 and 15) (Figure 3). Regarding miR-26a, its levels were higher in milk on Days 10 and 15 compared with the levels on Day 5 of lactation (repeated-measures ANOVA, post-hoc analysis). On Day 10, both the WD and Rev groups showed higher levels of miR-26a in the milk than controls (one-way ANOVA, post-hoc analysis). miR-125a levels increased in WD and Rev animals during lactation, but not in the controls (repeated-measures ANOVA, post-hoc analysis). On Day 15, both WD and Rev animals displayed greater levels of miR-125a in milk than the controls (one-way ANOVA, post-hoc analysis). miR-222 levels in milk increased progressively in all dams (repeated-measures ANOVA, post-hoc analysis). On Day 15 of lactation, WD animals displayed greater levels in milk than the controls (Mann–Whitney U-test). However, on Day 5, dams of the Rev group showed greater miR-222 levels than the controls, while levels in the WD group were not different from those of the control and Rev groups (one-way ANOVA, post-hoc analysis). Regarding miR-484, a different pattern was observed in the milk of the WD and Rev groups compared with the controls. miR-484 levels in the milk increased on Day 10 in all groups, compared with the levels on Day 5, and decreased on Day 15 in the controls but not in the WD and Rev groups (repeated-measures ANOVA, post-hoc analysis). On Day 15, Rev rats displayed greater miR-484 levels in milk, while the levels in WD animals were not different from those of the control and Rev groups (one-way ANOVA, post-hoc analysis).

**Figure 4:**
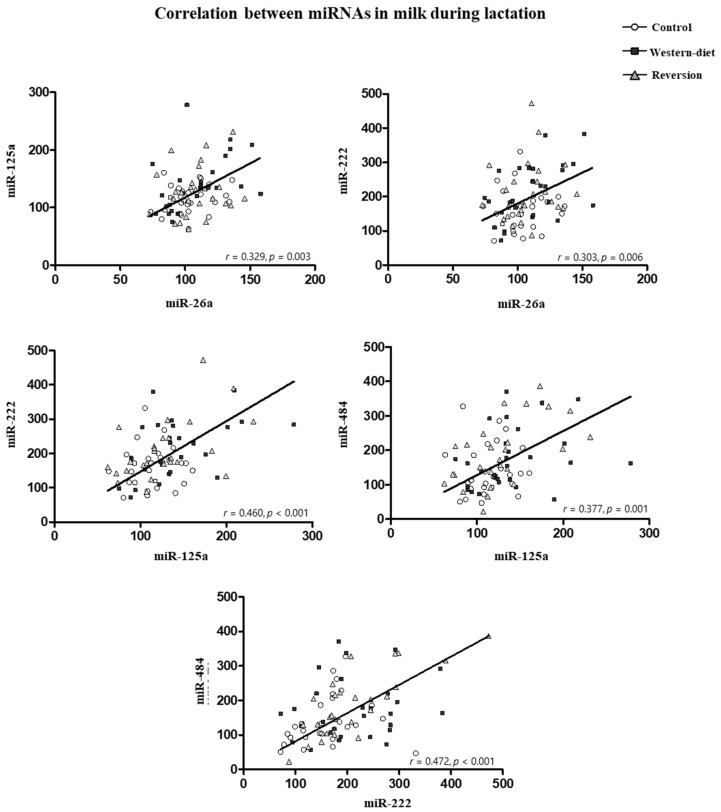
Correlation between miRNAs in milk during lactation. The correlations were assessed by Pearson’s correlation (2-tailed). Pearson’s correlation coefficients (*r*) and *p*-values are indicated.

The correlations among the miRNAs analyzed in milk that were significant (Pearson’s correlation) are shown in Figure 4. miR-222 correlated positively with miR-26a (r = 0.303, *p* = 0.006), miR-125a (r = 0.460, *p* < 0.001) and miR-484 (r = 0.472, *p* < 0.001). miR-125a also correlated positively with miR-26a (r = 0.329, *p* = 0.003) and miR-484 (r = 0.472, *p* < 0.001).

## 4. Discussion

MG development takes place from the fetal stage and continues during critical periods of life, such as pregnancy and lactation [40]. The intake of an obesogenic diet during lactation and/or maternal obesity have been shown to affect the development and function of the MG [15,41], in addition to having an impact on the metabolic health of the offspring [42]. However, the underlying mechanisms and whether such alterations can be prevented by improving maternal diet during lactation have not been fully elucidated. Considering that miRNA production by the MG may be modulated by maternal conditions [7,8,9], and that they may exert an effect on target mRNAs in the offspring [17,18], the present study aimed to investigate potential alterations in the levels of selected miRNAs in the MG and in the milk of diet-induced obese rats, and whether such changes could be prevented by dietary improvements during lactation.

We show here that obese rats on an obesogenic diet during lactation exhibited, at the end of lactation, greater expression levels of miR-26a, miR-222 and miR-484 in MG, but dietary improvement during lactation normalized their levels to those of the controls, despite these animals maintaining excess adiposity. It is also remarkable that these miRNAs were mutually correlated. Regarding miR-26a, its role in the MG has hardly been explored, while increased or decreased levels in different tissues have been linked to states of obesity and/or diabetes. Specifically, overweight humans and obese mouse models have been shown to exhibit decreased expression levels of miR-26a in the liver compared with lean individuals [43]. In fact, the silencing of endogenous miR-26a impairs insulin sensitivity, and enhances glucose production and fatty acid synthesis [43], whereas overexpression of miR-26a in mice fed a high-fat diet prevents obesity-induced metabolic complications, improves insulin sensitivity and decreases hepatic glucose production and fatty acid synthesis [43]. In contrast, mothers with Type 1 diabetes during lactation [44] and children with newly diagnosed Type 1 diabetes [28] display greater plasma miR-26a levels in comparison with their controls. Moreover, plasma miR-26a levels were positively correlated with glycated hemoglobin in children with Type 1 diabetes at diagnosis [45]. Here, we show that obese dams on an obesogenic diet (prior to mating and during gestation and lactation) (the WD group) displayed, at the end of lactation, greater miR-26a levels in the MG than the controls, and also in milk on Day 10. Of note, dietary improvements during lactation in obese rats (Rev group) normalized miR-26a levels in the MG but not their levels in milk. Unlike the present results, we previously described that nursing rats fed a cafeteria diet only during lactation displayed lower expression levels of miR-26a in the MG at the end of lactation compared with control dams, together with lower miR-26a levels in milk on Day 15 of lactation and no changes on Day 10 of lactation [17,18]. The differences in the results obtained regarding miR-26a levels in the milk between both studies could be attributed to the differences in the dietary intervention, particularly in terms of the period and type of intervention. In the present study, we evaluated changes in diet-induced obese rats, with or without Western diet exposure during lactation, whereas in the previous study [18], the cafeteria diet was offered only during lactation. Further research is needed to explain such differences among animal models, but these results suggest that the expression of miR-26a in the MG and its levels in milk are highly sensitive to maternal conditions, including diet and/or metabolic alterations.

To determine the functional significance of changes in the miR-26a levels in the MG, we studied the expression levels of validated target genes (*Insig1*, *Pten* and *Rb1* [17,34]) in the MG. Of interest, the greater levels of miR-26a in the MG of WD rats were accompanied by a decrease in the mRNA levels of *Rb1*. In addition, dietary improvement during lactation in Rev animals led to normalization of their expression levels, as well as an increase in the mRNA levels of *Insig1*, in comparison with the levels present in the WD group, which agreed with the normalization of miR-26a levels in Rev animals.

INSIG1 is a regulator of intracellular lipid metabolism, and abnormal expression of *Insig1* is widely involved in various lipid disorders [46]. Specifically, INSIG1 reduces the plasma levels of free cholesterol (by controlling the activation of sterol regulatory element-binding proteins and the degradation of 3-hydroxy-3-methylglutaryl-coenzyme A reductase) and protects β cells against lipotoxicity (by suppressing lipid droplet accumulation and fatty acid synthesis) [46]. Although the role of INSIG1 is well studied in hepatocytes and adipocytes, their role in MG is less known. In this regard, Wang et al. have described the functional relationship between the miR-26 family and its target gene, Insig1, in the regulation of milk fat synthesis in mammary epithelial cells in goats [34]. Moreover, overexpression of INSIG1 in mammary epithelial cells of buffalo has been associated with a reduction in the triglyceride content in these cells, suggesting a key role of INSIG1 in the regulation of milk fat synthesis in the MG [47]. Therefore, in the present study, the lower expression levels of *Insig1* in the MG of WD animals compared with the Rev group could be associated with a greater milk fat synthesis in the MG of obese dams that continued to be exposed to the obesogenic diet during lactation. In addition, the changes in the abundance of miR-26a were in accordance with the expression profile of another of its target genes, *Rb1*. The encoded protein, retinoblastoma Protein 1 (RB1), is a nuclear phosphoprotein that is critical in the regulation of cell cycle progression [48]. Therefore, the decreased *Rb1* mRNA levels found in the MG of obese dams exposed to an obesogenic diet during lactation, but not in obese dams after the dietary improvement, could tentatively affect cell proliferation capacity. This has not been directly assessed in these animals, but a trend toward a higher weight of the MG was observed in the WD rats compared with the controls (a 20% increase), although the results did not reach statistical significance (*p* = 0.110, Mann–Whitney U-test).

miR-222 levels in the MG were also increased in WD animals but were normalized in animals of the Rev group. Elevated miR-222 levels have been associated with obesity and/or diabetes, and with insulin resistance in both human and animal studies. Specifically, circulating miR-222 levels have been reported to be increased in obese and in Type 2 diabetic patients, while metformin treatment was shown to restore its levels [49,50,51,52]. Pregnant women with gestational diabetes mellitus (GDM) also displayed greater expression of miR-222 in omental adipose tissue [53], and greater plasma levels [54] versus control pregnant women. Using animal models, Ono et al. found that miR-222 levels were increased in the livers of mice fed a high-fat/high-sucrose diet, and this increase was associated with an impairment in insulin signaling [55]. In addition, overexpression of the miR-221/222 family was shown to impair insulin production and secretion by β-cells and resulted in glucose intolerance in mice [56]. Therefore, alterations in the levels of miR-222 in the MG may be related to alterations in insulin signaling. In fact, computational predictions of the target genes of the three miRNAs (miR-26a, miR-222 and miR-484) whose levels were altered in the WD group but not in the Rev group indicated a relevant role of these miRNAs modulating the expression of genes involved in insulin signaling. One of the validated target genes for miR-222 is the C-X-C chemokine receptor type 4 (Cxcr4) [37], a transmembrane receptor for C-X-C motif chemokine ligand 12 (CXCL12) [57]. The interaction between CXCL12 and its receptor, CXCR4, induces downstream signaling involved in chemotaxis, cell survival and proliferation [57]. Although the role of CXCL12 in diabetes is complex, the CXCL12/CXCR4 axis in adipose tissue has been associated with the production of proinflammatory cytokines and, finally, systemic insulin resistance [58]. It has also been reported that miR-222 levels were increased while *Cxcr4* mRNA levels were decreased in the placentas of women and mice with GDM [37]. Interestingly, the silencing of miR-222 was shown to suppress the inflammatory response and stimulate insulin sensitivity in mice with GDM by promoting the expression of *Cxcr4* [37]. Therefore, in the present study, it could be speculated that the presence of increased miR-222 levels in the MG of WD animals compared with both control and Rev animals, accompanied by the presence of lower mRNA *Cxcr4* levels in comparison with the Rev group, could contribute to a proinflammatory and insulin resistance state in these dams, and would be in accordance with the presence of elevated plasma insulin levels, as previously described [19]. Interestingly, the fact that miR-222 levels and the mRNA levels of its target gene *Cxcr4* were restored in the MG of obese dams fed an SD during lactation supports the relevance of this miRNA and interest in a nutritional intervention during lactation as a strategy to prevent such alterations in the MG associated with dietary obesity and, in turn, the proinflammatory and insulin-resistant states in these dams.

In line with what shown in the MG, the WD group also presented higher miR-222 levels in milk on Day 15 of lactation in comparison with their controls. This is in agreement with what was previously described in rats fed a cafeteria diet during lactation [18]. However, on Day 5 of lactation, the profile was somewhat different, with Rev animals showing higher miR-222 levels than the controls, and WD animals showing intermediate levels. Therefore, miR-222 levels in the milk appear to reflect both maternal diet and obesity status. In addition, it should also be highlighted that the secretion of this miRNA in the milk seems to be a time-dependent regulated process, since its levels increased progressively during lactation in all groups of dams, regardless of maternal conditions, which is consistent with our previous results [18].

miR-484 levels were also significantly higher in the MG of diet-induced obese dams that were maintained on a WD during lactation. Elevated serum levels of miR-484 have been described in individuals with coronary artery disease [59]. They were also upregulated in children with obesity compared with their normal-weight counterparts [60]. Computational functional analysis performed with miRNAs that were altered in WD dams has suggested a relevant role of the miRNAs modulating the expression of genes involved in the VEGFA-VEGFR2 signaling pathway, which is associated with obesity-related metabolic diseases [61]. Specifically, miR-484 has been shown to inhibit the expression of VEGF-A and VEGF-B [39]. VEGF-A is a growth factor that binds to members of the VEGF tyrosine kinase receptor (VEGFR) family on the surface of vascular endothelial cells to induce the proliferation and migration of vascular endothelial cells, and is essential for angiogenesis [61]. VEGF and its receptors have been shown to be upregulated in the MG during pregnancy and lactation [62,63]. This factor seems to be essential for MG differentiation and milk production [64]. Inactivation of VEGF in the MG epithelium in transgenic mice resulted in an impaired secretory activity of the epithelial cells, ultimately leading to reduced milk secretion and malnutrition in offspring [64]. Thus, VEGF must be considered as a factor that controls the correct function of the MG in the lactating state. However, as we show here, the production of this factor by the MG is decreased in obese dams, and its mRNA levels were not recovered by dietary normalization, despite the partial normalization of miR-484 levels in Rev animals. This could be tentatively related to the higher body fat content that Rev rats showed at the end of lactation compared with the controls, despite being on a SD. Notably, miR-484 levels in the milk also followed different patterns during lactation, since the levels experienced a peak on Day 10 of lactation in control rats, but the levels remained high in the WD and Rev groups. Therefore, the regulation of milk miR-484 levels and the expression of its target gene *Vegfa* in the MG appear to be associated primarily with excessive fat accumulation, rather than dietary conditions. However, more research is needed to further assess specific factors affecting the regulation of miR-484 and its target genes in the MG, and the potential consequences in the offspring.

Finally, WD animals displayed a greater miR-125a expression in MG compared with Rev animals. Circulating miR-125a levels have been found to be increased in hyperlipidemic and hyperglycemic patients [65]. In addition, miR-125a and miR-125b were overexpressed in the adipose tissue of obese patients with Type 2 diabetes compared with subjects with the same body mass index but without Type 2 diabetes [66]. Functional analysis indicated that miR-125a could negatively regulate elongase of very long chain fatty acids 6 (ELOVL6), which catalyzes the rate-limiting step in the elongation cycle, exerting a key function in milk fat synthesis [35,67]. Expression levels of the *Elovl6* gene in the MG have been shown to be increased during lactation as an adaptation to ensure the adequate concentration of long-chain polyunsaturated fatty acids required by the newborn [68]. Therefore, decreased expression of *Elovl6* in the MG of WD animals but not in Rev animals could be tentatively related with alterations in fatty acid metabolism in the MG, with potential consequences on the lipid composition of milk. However, despite the changes found in the expression of some of the target genes, a limitation of this study is that protein expression levels have not been measured. This analysis could provide relevant information, and deserves to be addressed in future studies.

In conclusion, here, we show a subset of miRNAs and target genes in the MG that could mediate, at least in part, alterations in lactating MG function due to maternal intake of an obesogenic diet. Interestingly, the implementation of a healthy diet during lactation in diet-induced obese rats attenuates most of these alterations, which highlights the importance of maternal diet during lactation. However, it is noteworthy that the dietary intervention did not fully normalize the altered miRNA levels in the milk. Further research is needed to better understand the determinants of the milk miRNA profile, in addition to the contribution of the MG, as well as to study the dynamics of the MG during lactation in relation to miRNA production.

## Figures and Tables

**Table 1:** Body weight and body fat content of the control, Western diet and reversion groups prior to gestation, on Day 17 of gestation and at the end of the lactation period. Data are expressed as the mean ± SEM of 8–10 animals per group. Statistics: MD, effect of maternal diet (one-way ANOVA). Least significant difference (LSD) post-hoc test, a ≠ b. These results were previously published in [19].

Phenotypic Traits		Control	Western Diet	Reversion	ANOVA
Prior to gestation	Body weight (g)	223 ± 9	253 ± 7	252 ± 11	-
	Body fat (g)	29.1 ± 2.1 a	51.5 ± 4.7 b	52.9 ± 6.6 b	MD
	Body fat (%)	12.4 ± 0.8 a	20.0 ± 1.3 b	20.4 ± 1.7 b	MD
Day 17 of gestation	Body weight (g)	309 ± 9	322 ± 8	324 ± 14	-
Day 21 of lactation	Body weight (g)	304 ± 9 a	283 ± 5 b	311 ± 7 a	MD
	Body fat (g)	29.5 ± 2.4 a	32.0 ± 2.2 a	41.3 ± 3.9 b	MD
	Body fat (%)	9.7 ± 0.8 a	11.3 ± 0.7 ab	13.2 ± 1 b	MD

**Table 2:** Altered WikiPathways based on the predicted target genes of miR-26a, miR-222 and miR-484. Detailed information on the GO terms, *p*-values after Bonferroni’s step-down correction and their associated genes (% and number) are indicated. The analysis was conducted using the ClueGo+Clupedia app in Cytoscape [32,33].

GO ID	GO Term	Corrected *p*-Value	% Associated Genes	No. Genes
WP:3888	VEGFA-VEGFR2 signaling pathway	0.0002	16.9	74
WP:2853	Endoderm differentiation	<0.0001	26.0	38
WP:2380	Brain-derived neurotrophic factor (BDNF) signaling pathway	<0.0001	25.0	36
WP:481	Insulin signaling	0.0003	22.4	36
WP:2857	Mesodermal commitment pathway	0.0004	22.3	35
WP:673	ErbB signaling pathway	0.0001	28.6	26
WP:4685	Melanoma	0.0002	30.9	21
WP:3657	Hematopoietic stem cell gene regulation by the GABP alpha/beta Complex	0.0004	50.0	11

## Supplementary Materials

The following supporting information can be downloaded at: https://www.mdpi.com/article/10.3390/biomedicines10061292/s1. Table S1: Nucleotide sequences of the primers. Table S2: List of putative target genes of miR-26a, miR-222 and miR-484 searched with TargetScan.
