# Supplementary material for: Dietary Improvement during Lactation Normalizes miR-26a, miR-222 and miR-484 Levels in the Mammary Gland, but Not in Milk, of Diet-Induced Obese Rats
Source: Biomedicines. 2022 May 31;10(6):1292. doi: 10.3390/biomedicines10061292 (PMC9219892; doi:10.3390/biomedicines10061292)
Supplement: Supplementary file 1 [file biomedicines-10-01292-s001.zip › biomedicines-1739674-supplementary.pdf]

Table S1. Nucleotide sequences of primers.

Gene	Forward Primer (5' to 3')	Reverse Primer (5' to 3')	Amplicon Size (pb)
Gdi	CCGCACAAGGCAAATACATC	GACTCTCTGAACCGTCATCAA	210
Insig1	ATCACCATCGCCTTCCTAGC	TGTTTCCCACTGTGACACCT	142
Pten	CAGAAAAAGTGGAAAATGGAAG	TGGAGAGAAGTATCGGTTGG	158
Rb1	CTCACGCTGCCCAGGAGACC	GTAGGAGGCCTGGTGGAGGCA	139
Elovl6	AGAACACTGCTGCTGAGGGA	GTCCACTAATCTCCTTGCCCA	95
Stat3	GCTGACCAATAACCCCAAGA	ACACCCTGAGTAGTTCACACCA	181
Cdkn1b	CAGAATCATAAGCCCCTGGA	GACGAGTCAGGCATTTGGTC	221
Cxcr4	GCCATGGCTGACTGGTACTT	CACCCACATAGACGGCCTTT	187
Vegfa	TCGGAGAGCAACGTCACTATGCA	GGCTCACAGTGAACGCTCCAGG	215

Table S2. List of putative target genes of miR-26a, miR-222 and miR-484 searched with TargetScan.

Gene Symbol	Gene Name	Target miRNA
A1CF	APOBEC1 complementation factor	miR-26a
AAK1	AP2 associated kinase 1	miR-222
AAR2	AAR2 splicing factor homolog (S. cerevisiae)	mir-484
ABCA1	ATP-binding cassette, sub-family A (ABC1), member 1	miR-26a
ABCC4	ATP-binding cassette, sub-family C (CFTR/MRP), member 4	miR-26a
ABHD12	abhydrolase domain containing 12	mir-484
ABHD2	abhydrolase domain containing 2	miR-26a; miR-484
ABHD5	abhydrolase domain containing 5	miR-26a
ABI2	abl-interactor 2	miR-26a
ABL2	c-abl oncogene 2, non-receptor tyrosine kinase	miR-26a
ACADSB	acyl-CoA dehydrogenase, short/branched chain	miR-26a
ACBD5	acyl-CoA binding domain containing 5	miR-26a
ACSL3	acyl-CoA synthetase long-chain family member 3	miR-26a
ACTR3	ARP3 actin-related protein 3 homolog (yeast)	mir-484
ACVR1B	activin A receptor, type IB	mir-484
ACVR1C	activin A receptor, type IC	miR-26a; miR-484
ACVR2B	activin A receptor, type IIB	miR-26a; miR-222
ADAM10	ADAM metallopeptidase domain 10	miR-26a
ADAM11	ADAM metallopeptidase domain 11	miR-222
ADAM12	ADAM metallopeptidase domain 12	miR-26a
ADAM17	ADAM metallopeptidase domain 17	miR-26a
ADAM19	ADAM metallopeptidase domain 19	miR-26a
ADAM22	ADAM metallopeptidase domain 22	mir-484
ADAM23	ADAM metallopeptidase domain 23	miR-26a
ADAMTS19	ADAM metallopeptidase with thrombospondin type 1 motif, 19	miR-26a
ADAMTS5	ADAM metallopeptidase with thrombospondin type 1 motif, 5	miR-26a

ADARB1	adenosine deaminase, RNA-specific, B1	mir-484
ADD2	adducin 2 (beta)	mir-484
ADM	adrenomedullin	miR-26a
ADNP	activity-dependent neuroprotector homeobox	mir-484
ADO	2-aminoethanethiol (cysteamine) dioxygenase	mir-484
ADORA1	adenosine A1 receptor	mir-484
AGAP2	ArfGAP with GTPase domain, ankyrin repeat and PH domain 2	mir-484
AGFG1	ArfGAP with FG repeats 1	miR-222
AGFG2	ArfGAP with FG repeats 2	miR-222
AGL	amylo-alpha-1, 6-glucosidase, 4-alpha-glucanotrans-ferase	mir-484
AGPAT2	1-acylglycerol-3-phosphate O-acyltransferase 2	mir-484
AGPAT3	1-acylglycerol-3-phosphate O-acyltransferase 3	miR-26a
AGPS	alkylglycerone phosphate synthase	miR-222
AJAP1	adherens junctions associated protein 1	miR-222
AK4	adenylate kinase 4	mir-484
AKIRIN1	akirin 1	miR-26a
ALDH5A1	aldehyde dehydrogenase 5 family, member A1	miR-26a
ALKBH5	alkB, alkylation repair homolog 5 (E. coli)	mir-484
ALKBH6	alkB, alkylation repair homolog 6 (E. coli)	mir-484
AMIGO1	adhesion molecule with Ig-like domain 1	miR-222
AMMECR1	Alport syndrome, mental retardation, midface hypo-plasia and elliptocytosis chromosomal region gene 1	miR-26a; miR-222
AMOT	angiomotin	miR-26a
ANAPC16	anaphase promoting complex subunit 16	miR-222
ANGPTL2	angiopoietin-like 2	miR-222
ANK2	ankyrin 2, neuronal	miR-26a
ANKHD1	ankyrin repeat and KH domain containing 1	miR-222
ANKIB1	ankyrin repeat and IBR domain containing 1	miR-26a; miR-222
ANKRD10	ankyrin repeat domain 10	mir-484
ANKRD11	ankyrin repeat domain 11	miR-26a
ANKRD13A	ankyrin repeat domain 13A	mir-484
ANKRD27	ankyrin repeat domain 27 (VPS9 domain)	mir-484
ANKRD52	ankyrin repeat domain 52	miR-26a; miR-222
ANKRD63	ankyrin repeat domain 63	miR-26a
ANKRD9	ankyrin repeat domain 9	mir-484
ANKS1A	ankyrin repeat and sterile alpha motif domain contain-ing 1A	miR-26a
ANKS1B	ankyrin repeat and sterile alpha motif domain contain-ing 1B	miR-26a
ANKS4B	ankyrin repeat and sterile alpha motif domain contain-ing 4B	mir-484
AP3M1	adaptor-related protein complex 3, mu 1 subunit	miR-222
AP4E1	adaptor-related protein complex 4, epsilon 1 subunit	mir-484
AP5M1	adaptor-related protein complex 5, mu 1 subunit	miR-26a

APC	adenomatous polyposis coli	miR-26a
APEH	acylaminoacyl-peptide hydrolase	mir-484
APLNR	apelin receptor	mir-484
APOOL	apolipoprotein O-like	miR-26a
APP	amyloid beta (A4) precursor protein	miR-26a
AR	androgen receptor	mir-484
ARF4	ADP-ribosylation factor 4	miR-222
ARF6	ADP-ribosylation factor 6	miR-26a
ARFGEF1	ADP-ribosylation factor guanine nucleotide-exchange factor 1 (brefeldin A-inhibited)	miR-26a
ARHGAP19	Rho GTPase activating protein 19	mir-484
ARHGAP21	Rho GTPase activating protein 21	miR-26a
ARHGAP26	Rho GTPase activating protein 26	miR-26a
ARHGAP42	Rho GTPase activating protein 42	miR-222
ARHGDIA	Rho GDP dissociation inhibitor (GDI) alpha	mir-484
ARHGEF10	Rho guanine nucleotide exchange factor (GEF) 10	miR-26a
ARHGEF12	Rho guanine nucleotide exchange factor (GEF) 12	miR-26a
ARHGEF25	Rho guanine nucleotide exchange factor (GEF) 25	mir-484
ARHGEF26	Rho guanine nucleotide exchange factor (GEF) 26	miR-26a
ARHGEF6	Rac/Cdc42 guanine nucleotide exchange factor (GEF) 6	mir-484
ARID1A	AT rich interactive domain 1A (SWI-like)	miR-222
ARID2	AT rich interactive domain 2 (ARID, RFX-like)	miR-26a
ARID3A	AT rich interactive domain 3A (BRIGHT-like)	miR-26a
ARID3B	AT rich interactive domain 3B (BRIGHT-like)	miR-222
ARL4C	ADP-ribosylation factor-like 4C	miR-26a
ARL6IP6	ADP-ribosylation-like factor 6 interacting protein 6	miR-26a
ARMCX2	armadillo repeat containing, X-linked 2	miR-26a
ARNT	aryl hydrocarbon receptor nuclear translocator	miR-222
ARNTL2	aryl hydrocarbon receptor nuclear translocator-like 2	mir-484
ARPC3	actin related protein 2/3 complex, subunit 3, 21kDa	mir-484
ARRB1	arrestin, beta 1	mir-484
ARSI	arylsulfatase family, member I	mir-484
ARTN	artemin	mir-484
ASB1	ankyrin repeat and SOCS box containing 1	mir-484
ASCL2	achaete-scute complex homolog 2 (Drosophila)	miR-26a
ASPN	asporin	miR-26a
ATAD2B	ATPase family, AAA domain containing 2B	miR-26a; miR-222
ATF2	activating transcription factor 2	miR-26a; miR-484
ATF5	activating transcription factor 5	mir-484
ATL3	atlastin GTPase 3	miR-26a
ATP11C	ATPase, class VI, type 11C	miR-26a
ATP1A2	ATPase, Na+/K+ transporting, alpha 2 polypeptide	miR-26a
ATP1B1	ATPase, Na+/K+ transporting, beta 1 polypeptide	miR-222
ATP1B4	ATPase, Na+/K+ transporting, beta 4 polypeptide	miR-26a

ATP2A3	ATPase, Ca++ transporting, ubiquitous	mir-484
ATP6V1D	ATPase, H+ transporting, lysosomal 34kDa, V1 subunit D	mir-484
ATP7A	ATPase, Cu++ transporting, alpha polypeptide	mir-484
ATP8A2	ATPase, aminophospholipid transporter, class I, type 8A, member 2	miR-222
ATPAF1	ATP synthase mitochondrial F1 complex assembly factor 1	miR-26a
ATXN1	ataxin 1	miR-222
ATXN7	ataxin 7	miR-26a; miR-222
ATXN7L1	ataxin 7-like 1	mir-484
AXIN2	axin 2	miR-222
AZI2	5-azacytidine induced 2	miR-26a
B4GALT1	UDP-Gal:betaGlcNAc beta 1,4- galactosyltransferase, polypeptide 1	miR-26a
BABAM1	BRISC and BRCA1 A complex member 1	mir-484
BAHD1	bromo adjacent homology domain containing 1	mir-484
BAK1	BCL2-antagonist/killer 1	miR-26a
BARX2	BARX homeobox 2	mir-484
BAZ2A	bromodomain adjacent to zinc finger domain, 2A	mir-484
BAZ2B	bromodomain adjacent to zinc finger domain, 2B	miR-26a; miR-222
BBC3	BCL2 binding component 3	miR-222
BBX	bobby sox homolog (Drosophila)	miR-26a
BCKDHB	branched chain keto acid dehydrogenase E1, beta polypeptide	mir-484
BCL2	B-cell CLL/lymphoma 2	mir-484
BCL2L1	BCL2-like 1	mir-484
BCL2L11	BCL2-like 11 (apoptosis facilitator)	miR-222
BCL2L13	BCL2-like 13 (apoptosis facilitator)	mir-484
BCL2L2	BCL2-like 2	mir-484
BCR	breakpoint cluster region	miR-26a
BEND4	BEN domain containing 4	miR-222; miR-484
BFAR	bifunctional apoptosis regulator	miR-26a
BHLHE22	basic helix-loop-helix family, member e22	miR-26a
BHLHE40	basic helix-loop-helix family, member e40	miR-26a
BHLHE41	basic helix-loop-helix family, member e41	miR-26a
BICD2	bicaudal D homolog 2 (Drosophila)	miR-26a
BLOC1S2	biogenesis of lysosomal organelles complex-1, subunit 2	miR-26a
BMF	Bcl2 modifying factor	miR-222
BMP2K	BMP2 inducible kinase	miR-26a
BMP8A	bone morphogenetic protein 8a	mir-484
BOD1	biorientation of chromosomes in cell division 1	miR-26a
BRAP	BRCA1 associated protein	miR-26a
BRD4	bromodomain containing 4	mir-484

BRWD1	bromodomain and WD repeat domain containing 1	miR-26a; miR-222
BRWD3	bromodomain and WD repeat domain containing 3	miR-26a; miR-222
BTBD3	BTB (POZ) domain containing 3	mir-484
BTBD7	BTB (POZ) domain containing 7	miR-26a
BTG1	B-cell translocation gene 1, anti-proliferative	miR-26a
BTG2	BTG family, member 2	miR-222
BTG4	B-cell translocation gene 4	mir-484
BYSL	bystin-like	miR-222
BZRAP1	benzodiazapine receptor (peripheral) associated protein 1	mir-484
BZW1	basic leucine zipper and W2 domains 1	miR-222
C10orf12	chromosome 10 open reading frame 12	miR-26a
C10orf137	chromosome 10 open reading frame 137	miR-26a
C11orf30	chromosome 11 open reading frame 30	mir-484
C11orf87	chromosome 11 open reading frame 87	miR-222
C14orf105	chromosome 14 open reading frame 105	mir-484
C15orf27	chromosome 15 open reading frame 27	mir-484
C15orf32	chromosome 15 open reading frame 32	mir-484
C15orf61	chromosome 15 open reading frame 61	miR-26a
C15orf62	chromosome 15 open reading frame 62	mir-484
C16orf52	chromosome 16 open reading frame 52	miR-222
C16orf58	chromosome 16 open reading frame 58	mir-484
C16orf70	chromosome 16 open reading frame 70	miR-26a
C16orf92	chromosome 16 open reading frame 92	miR-222
C17orf59	chromosome 17 open reading frame 59	mir-484
C17orf78	chromosome 17 open reading frame 78	mir-484
C17orf80	chromosome 17 open reading frame 80	mir-484
C18orf25	chromosome 18 open reading frame 25	miR-26a; miR-222
C19orf26	chromosome 19 open reading frame 26	mir-484
C1GALT1	core 1 synthase, glycoprotein-N-acetylgalactosamine 3-beta-galactosyltransferase, 1	miR-26a
C1orf116	chromosome 1 open reading frame 116	mir-484
C1orf220	chromosome 1 open reading frame 220	mir-484
C1orf95	chromosome 1 open reading frame 95	mir-484
C1RL	complement component 1, r subcomponent-like	mir-484
C20orf196	chromosome 20 open reading frame 196	mir-484
C20orf24	chromosome 20 open reading frame 24	miR-26a
C2CD2L	C2CD2-like	miR-26a
C2CD5	C2 calcium-dependent domain containing 5	miR-26a
C2orf88	chromosome 2 open reading frame 88	mir-484
C3orf62	chromosome 3 open reading frame 62	mir-484
C3orf70	chromosome 3 open reading frame 70	miR-222
C5orf63	chromosome 5 open reading frame 63	mir-484
C7orf41	chromosome 7 open reading frame 41	mir-484

C8orf46	chromosome 8 open reading frame 46	mir-484
C9orf129	chromosome 9 open reading frame 129	mir-484
C9orf139	chromosome 9 open reading frame 139	mir-484
C9orf47	chromosome 9 open reading frame 47	mir-484
C9orf64	chromosome 9 open reading frame 64	mir-484
CABP7	calcium binding protein 7	miR-222; miR-484
CACFD1	calcium channel flower domain containing 1	mir-484
CACNA1C	calcium channel, voltage-dependent, L type, alpha 1C subunit	miR-26a
CACNA1E	calcium channel, voltage-dependent, R type, alpha 1E subunit	mir-484
CACNA1H	calcium channel, voltage-dependent, T type, alpha 1H subunit	mir-484
CACNA1I	calcium channel, voltage-dependent, T type, alpha 1I subunit	mir-484
CACNB4	calcium channel, voltage-dependent, beta 4 subunit	miR-26a; miR-222
CAMK2A	calcium/calmodulin-dependent protein kinase II alpha	miR-26a
CAMKK1	calcium/calmodulin-dependent protein kinase kinase 1, alpha	miR-222
CAMKK2	calcium/calmodulin-dependent protein kinase kinase 2, beta	miR-26a
CAMSAP1	calmodulin regulated spectrin-associated protein 1	miR-26a
CANX	calnexin	miR-222
CAPN7	calpain 7	mir-484
CAPRIN2	caprin family member 2	miR-222
CAPZA1	capping protein (actin filament) muscle Z-line, alpha 1	miR-26a
CAPZB	capping protein (actin filament) muscle Z-line, beta	miR-26a
CARM1	coactivator-associated arginine methyltransferase 1	miR-26a
CASZ1	castor zinc finger 1	miR-26a; miR-222
CATSPER2	cation channel, sperm associated 2	mir-484
CBFB	core-binding factor, beta subunit	miR-222
CBL	Cbl proto-oncogene, E3 ubiquitin protein ligase	miR-222
CBS	cystathionine-beta-synthase	mir-484
CBX5	chromobox homolog 5	mir-484
CBX8	chromobox homolog 8	mir-484
CC2D1B	coiled-coil and C2 domain containing 1B	miR-222
CCAR2	cell cycle and apoptosis regulator 2	miR-26a
CCDC102A	coiled-coil domain containing 102A	mir-484
CCDC103	coiled-coil domain containing 103	mir-484
CCDC117	coiled-coil domain containing 117	mir-484
CCDC171	coiled-coil domain containing 171	miR-26a; miR-222
CCDC28A	coiled-coil domain containing 28A	miR-26a
CCDC36	coiled-coil domain containing 36	mir-484
CCDC37	coiled-coil domain containing 37	mir-484

CCDC42	coiled-coil domain containing 42	mir-484
CCDC50	coiled-coil domain containing 50	miR-222
CCDC6	coiled-coil domain containing 6	miR-26a
CCDC64	coiled-coil domain containing 64	miR-222
CCND2	cyclin D2	miR-26a
CCNJ	cyclin J	miR-26a
CCNJL	cyclin J-like	miR-26a
CCPG1	cell cycle progression 1	mir-484
CCSER2	coiled-coil serine-rich protein 2	miR-26a
CD200	CD200 molecule	miR-26a
CD2AP	CD2-associated protein	miR-222
CD4	CD4 molecule	miR-222; miR-484
CD72	CD72 molecule	mir-484
CD93	CD93 molecule	mir-484
CDC37L1	cell division cycle 37-like 1	mir-484
CDC42BPB	CDC42 binding protein kinase beta (DMPK-like)	mir-484
CDC42BPG	CDC42 binding protein kinase gamma (DMPK-like)	mir-484
CDC7	cell division cycle 7	mir-484
CDC73	cell division cycle 73	miR-26a
CDCP1	CUB domain containing protein 1	mir-484
CDH11	cadherin 11, type 2, OB-cadherin (osteoblast)	miR-26a
CDH19	cadherin 19, type 2	mir-484
CDH2	cadherin 2, type 1, N-cadherin (neuronal)	miR-26a; miR-222
CDH20	cadherin 20, type 2	miR-26a
CDH4	cadherin 4, type 1, R-cadherin (retinal)	miR-26a; miR-222
CDH6	cadherin 6, type 2, K-cadherin (fetal kidney)	mir-484
CDHR3	cadherin-related family member 3	mir-484
CDIP1	cell death-inducing p53 target 1	mir-484
CDK12	cyclin-dependent kinase 12	mir-484
CDK13	cyclin-dependent kinase 13	miR-26a
CDK2AP1	cyclin-dependent kinase 2 associated protein 1	miR-26a
CDK6	cyclin-dependent kinase 6	miR-26a
CDK8	cyclin-dependent kinase 8	miR-26a; miR-222
CDKL2	cyclin-dependent kinase-like 2 (CDC2-related kinase)	mir-484
CDKN1B	cyclin-dependent kinase inhibitor 1B (p27, Kip1)	miR-222
CDKN1C	cyclin-dependent kinase inhibitor 1C (p57, Kip2)	miR-222
CDR1	cerebellar degeneration-related protein 1, 34kDa	mir-484
CDR2L	cerebellar degeneration-related protein 2-like	miR-26a
CEBPG	CCAAT/enhancer binding protein (C/EBP), gamma	miR-26a
CELF1	CUGBP, Elav-like family member 1	miR-26a
CELF2	CUGBP, Elav-like family member 2	miR-26a
CELSR1	cadherin, EGF LAG seven-pass G-type receptor 1	miR-26a
CEND1	cell cycle exit and neuronal differentiation 1	mir-484

CENPB	centromere protein B, 80kDa	mir-484
CEP350	centrosomal protein 350kDa	miR-26a
CEP85	centrosomal protein 85kDa	mir-484
CFL1	cofilin 1 (non-muscle)	mir-484
CHAC1	ChaC, cation transport regulator homolog 1 (E. coli)	miR-26a
CHD1	chromodomain helicase DNA binding protein 1	miR-26a
CHD2	chromodomain helicase DNA binding protein 2	miR-222; miR-484
CHD3	chromodomain helicase DNA binding protein 3	mir-484
CHD7	chromodomain helicase DNA binding protein 7	miR-222
CHD8	chromodomain helicase DNA binding protein 8	miR-222
CHFR	checkpoint with forkhead and ring finger domains, E3 ubiquitin protein ligase	miR-26a
CHIC1	cysteine-rich hydrophobic domain 1	miR-26a
CHORDC1	cysteine and histidine-rich domain (CHORD) containing 1	miR-26a
CHRND	cholinergic receptor, nicotinic, delta (muscle)	mir-484
CHST2	carbohydrate (N-acetylglucosamine-6-O) sulfotransferase 2	miR-26a
CHSY1	chondroitin sulfate synthase 1	miR-26a; miR-222
CIB2	calcium and integrin binding family member 2	mir-484
CISD3	CDGSH iron sulfur domain 3	miR-26a
CKS2	CDC28 protein kinase regulatory subunit 2	miR-26a
CLASP2	cytoplasmic linker associated protein 2	miR-26a
CLCN5	chloride channel, voltage-sensitive 5	mir-484
CLDN20	claudin 20	mir-484
CLEC4F	C-type lectin domain family 4, member F	mir-484
CLOCK	clock circadian regulator	mir-484
CLRN1	clarin 1	mir-484
CLVS2	clavesin 2	miR-222; miR-484
CLYBL	citrate lyase beta like	mir-484
CMPK2	cytidine monophosphate (UMP-CMP) kinase 2, mitochondrial	mir-484
CMTM4	CKLF-like MARVEL transmembrane domain containing 4	miR-26a; miR-222
CMTM6	CKLF-like MARVEL transmembrane domain containing 6	mir-484
CMTR2	cap methyltransferase 2	mir-484
CNIH1	cornichon family AMPA receptor auxiliary protein 1	miR-26a
CNKSR2	connector enhancer of kinase suppressor of Ras 2	mir-484
CNNM2	cyclin M2	miR-26a
CNOT2	CCR4-NOT transcription complex, subunit 2	miR-222
CNPY1	canopy FGF signaling regulator 1	mir-484
CNR1	cannabinoid receptor 1 (brain)	mir-484
CNTFR	ciliary neurotrophic factor receptor	mir-484
COL10A1	collagen, type X, alpha 1	miR-26a

COL11A1	collagen, type XI, alpha 1	miR-26a
COL19A1	collagen, type XIX, alpha 1	miR-26a
COL1A2	collagen, type I, alpha 2	miR-26a
COL22A1	collagen, type XXII, alpha 1	miR-26a
COL5A1	collagen, type V, alpha 1	miR-26a
COPS2	COP9 signalosome subunit 2	miR-26a
COPS7B	COP9 signalosome subunit 7B	miR-26a
COX5A	cytochrome c oxidase subunit Va	miR-26a
CPEB2	cytoplasmic polyadenylation element binding protein 2	miR-26a
CPEB3	cytoplasmic polyadenylation element binding protein 3	miR-26a; miR-222
CPEB4	cytoplasmic polyadenylation element binding protein 4	miR-26a
CPED1	cadherin-like and PC-esterase domain containing 1	miR-26a
CPNE8	copine VIII	miR-222
CPPED1	calcineurin-like phosphoesterase domain containing 1	miR-222
CPSF2	cleavage and polyadenylation specific factor 2, 100kDa	miR-26a
CREB1	cAMP responsive element binding protein 1	miR-26a
CREBBP	CREB binding protein	miR-26a
CREBRF	CREB3 regulatory factor	miR-26a
CREBZF	CREB/ATF bZIP transcription factor	miR-26a; miR-222
CRK	v-crk avian sarcoma virus CT10 oncogene homolog	miR-222
CRLF3	cytokine receptor-like factor 3	miR-26a
CRTC2	CREB regulated transcription coactivator 2	mir-484
CRTC3	CREB regulated transcription coactivator 3	mir-484
CSF1R	colony stimulating factor 1 receptor	mir-484
CSNK1G1	casein kinase 1, gamma 1	miR-26a
CSRNP3	cysteine-serine-rich nuclear protein 3	mir-484
CT62	cancer/testis antigen 62	mir-484
CTCF	CCCTC-binding factor (zinc finger protein)	miR-222
CTDSP2	CTD (carboxy-terminal domain, RNA polymerase II, polypeptide A) small phosphatase 2	miR-26a
CTDSPL	CTD (carboxy-terminal domain, RNA polymerase II, polypeptide A) small phosphatase-like	mir-484
CTGF	connective tissue growth factor	miR-26a
CTIF	CBP80/20-dependent translation initiation factor	miR-222
CTNNBIP1	catenin, beta interacting protein 1	miR-26a
CTNND2	catenin (cadherin-associated protein), delta 2	miR-26a
CTTN	cortactin	miR-222
CTTNBP2NL	CTTNBP2 N-terminal like	miR-26a
CXCR3	chemokine (C-X-C motif) receptor 3	mir-484
CXCR5	chemokine (C-X-C motif) receptor 5	miR-222
CXorf23	chromosome X open reading frame 23	miR-26a
CYB5RL	cytochrome b5 reductase-like	mir-484

CYP2A6	cytochrome P450, family 2, subfamily A, polypeptide 6	mir-484
CYP2A7	cytochrome P450, family 2, subfamily A, polypeptide 7	mir-484
CYP2W1	cytochrome P450, family 2, subfamily W, polypeptide 1	mir-484
CYR61	cysteine-rich, angiogenic inducer, 61	miR-222
DAB2	Dab, mitogen-responsive phosphoprotein, homolog 2 (Drosophila)	miR-26a
DAPK1	death-associated protein kinase 1	miR-26a
DBNDD2	dysbindin (dystrobrevin binding protein 1) domain containing 2	mir-484
DBNL	drebrin-like	miR-222
DCAF11	DDB1 and CUL4 associated factor 11	mir-484
DCAF7	DDB1 and CUL4 associated factor 7	miR-26a; miR-222
DCBLD1	discoidin, CUB and LCCL domain containing 1	miR-26a
DCBLD2	discoidin, CUB and LCCL domain containing 2	miR-26a
DCDC2	doublecortin domain containing 2	miR-26a
DCP1A	decapping mRNA 1A	mir-484
DCUN1D1	DCN1, defective in cullin neddylation 1, domain containing 1	miR-222
DCUN1D4	DCN1, defective in cullin neddylation 1, domain containing 4	miR-222
DCUN1D5	DCN1, defective in cullin neddylation 1, domain containing 5	miR-26a
DDIT4	DNA-damage-inducible transcript 4	miR-222
DDR1	discoidin domain receptor tyrosine kinase 1	mir-484
DDR2	discoidin domain receptor tyrosine kinase 2	miR-26a
DDT	D-dopachrome tautomerase	mir-484
DDX17	DEAD (Asp-Glu-Ala-Asp) box helicase 17	miR-26a
DDX3X	DEAD (Asp-Glu-Ala-Asp) box helicase 3, X-linked	miR-26a
DDX3Y	DEAD (Asp-Glu-Ala-Asp) box helicase 3, Y-linked	miR-26a
DDX54	DEAD (Asp-Glu-Ala-Asp) box polypeptide 54	mir-484
DENND2C	DENN/MADD domain containing 2C	mir-484
DENND5A	DENN/MADD domain containing 5A	mir-484
DEPDC1B	DEP domain containing 1B	miR-26a
DERL2	derlin 2	miR-26a
DGCR14	DiGeorge syndrome critical region gene 14	miR-26a; miR-484
DGCR2	DiGeorge syndrome critical region gene 2	mir-484
DGCR8	DGCR8 microprocessor complex subunit	mir-484
DGKD	diacylglycerol kinase, delta 130kDa	mir-484
DGKG	diacylglycerol kinase, gamma 90kDa	mir-484
DGKH	diacylglycerol kinase, eta	miR-26a; miR-222
DGKZ	diacylglycerol kinase, zeta	mir-484
DHX36	DEAH (Asp-Glu-Ala-His) box polypeptide 36	miR-222

DHX57	DEAH (Asp-Glu-Ala-Asp/His) box polypeptide 57	mir-484
DIDO1	death inducer-obliterator 1	miR-26a
DIEXF	digestive organ expansion factor homolog (zebrafish)	mir-484
DIO3	deiodinase, iodothyronine, type III	mir-484
DIRAS1	DIRAS family, GTP-binding RAS-like 1	mir-484
DKK2	dickkopf WNT signaling pathway inhibitor 2	miR-222
DLEC1	deleted in lung and esophageal cancer 1	mir-484
DLG2	discs, large homolog 2 (Drosophila)	miR-222
DLG4	discs, large homolog 4 (Drosophila)	miR-26a
DLG5	discs, large homolog 5 (Drosophila)	miR-26a
DMD	dystrophin	miR-26a
DMRT3	doublesex and mab-3 related transcription factor 3	miR-26a; miR-222
DMXL1	Dmx-like 1	miR-26a
DNAH10OS	dynein, axonemal, heavy chain 10 opposite strand	miR-26a
DNAJA2	DnaJ (Hsp40) homolog, subfamily A, member 2	miR-26a
DNAJC14	DnaJ (Hsp40) homolog, subfamily C, member 14	miR-222
DNAJC21	DnaJ (Hsp40) homolog, subfamily C, member 21	miR-26a
DNAJC24	DnaJ (Hsp40) homolog, subfamily C, member 24	mir-484
DNAL1	dynein, axonemal, light chain 1	miR-222
DND1	DND microRNA-mediated repression inhibitor 1	mir-484
DNM1L	dynamin 1-like	miR-26a; miR-484
DNMT3A	DNA (cytosine-5-)-methyltransferase 3 alpha	miR-26a
DNPEP	aspartyl aminopeptidase	mir-484
DOCK4	dedicator of cytokinesis 4	miR-26a
DOCK9	dedicator of cytokinesis 9	mir-484
DPH3	diphthamide biosynthesis 3	mir-484
DRAM1	DNA-damage regulated autophagy modulator 1	miR-26a
DRD5	dopamine receptor D5	mir-484
DST	dystonin	miR-26a
DTNA	dystrobrevin, alpha	mir-484
DUSP5	dual specificity phosphatase 5	miR-26a
DYRK1A	dual-specificity tyrosine-(Y)-phosphorylation regulated kinase 1A	miR-26a; miR-222
E2F2	E2F transcription factor 2	miR-222
E2F7	E2F transcription factor 7	miR-26a
EAF1	ELL associated factor 1	miR-26a
ECHDC3	enoyl CoA hydratase domain containing 3	mir-484
EDN2	endothelin 2	mir-484
EEF2K	eukaryotic elongation factor-2 kinase	mir-484
EFCAB11	EF-hand calcium binding domain 11	mir-484
EFNB1	ephrin-B1	mir-484
EFNB2	ephrin-B2	mir-484
EFS	embryonal Fyn-associated substrate	mir-484
EHD1	EH-domain containing 1	miR-26a

EIF2S1	eukaryotic translation initiation factor 2, subunit 1 alpha, 35kDa	miR-26a
EIF3A	eukaryotic translation initiation factor 3, subunit A	miR-26a
EIF3J	eukaryotic translation initiation factor 3, subunit J	miR-222
EIF4E3	eukaryotic translation initiation factor 4E family member 3	miR-222
EIF4G2	eukaryotic translation initiation factor 4 gamma, 2	miR-26a
EIF5	eukaryotic translation initiation factor 5	miR-26a
EIF5A2	eukaryotic translation initiation factor 5A2	miR-222
ELAVL2	ELAV like neuron-specific RNA binding protein 2	miR-26a; miR-222
ELAVL3	ELAV like neuron-specific RNA binding protein 3	miR-26a
ELFN2	extracellular leucine-rich repeat and fibronectin type III domain containing 2	mir-484
ELK1	ELK1, member of ETS oncogene family	mir-484
ELP4	elongator acetyltransferase complex subunit 4	miR-222
EMP2	epithelial membrane protein 2	mir-484
EMX1	empty spiracles homeobox 1	mir-484
ENC1	ectodermal-neural cortex 1 (with BTB domain)	miR-26a
ENOX1	ecto-NOX disulfide-thiol exchanger 1	miR-26a
ENPP5	ectonucleotide pyrophosphatase/phosphodiesterase 5 (putative)	mir-484
ENTPD7	ectonucleoside triphosphate diphosphohydrolase 7	miR-26a
EOMES	eomesodermin	mir-484
EP300	E1A binding protein p300	miR-26a
EP400	E1A binding protein p400	miR-26a
EPAS1	endothelial PAS domain protein 1	miR-26a
EPB41L3	erythrocyte membrane protein band 4.1-like 3	miR-26a
EPC1	enhancer of polycomb homolog 1 (Drosophila)	miR-26a
EPC2	enhancer of polycomb homolog 2 (Drosophila)	miR-26a
EPG5	ectopic P-granules autophagy protein 5 homolog (C. elegans)	miR-26a
EPGN	epithelial mitogen	mir-484
EPHA2	EPH receptor A2	miR-26a
EPHA5	EPH receptor A5	miR-26a
EPHA7	EPH receptor A7	miR-26a
EPS15	epidermal growth factor receptor pathway substrate 15	miR-26a; miR-484
ERBB3	v-erb-b2 avian erythroblastic leukemia viral oncogene homolog 3	mir-484
ERBB4	v-erb-b2 avian erythroblastic leukemia viral oncogene homolog 4	miR-26a; miR-222
ERC2	ELKS/RAB6-interacting/CAST family member 2	miR-26a
ERLIN1	ER lipid raft associated 1	miR-26a
ERN2	endoplasmic reticulum to nucleus signaling 2	mir-484
ERO1LB	ERO1-like beta (S. cerevisiae)	miR-26a; miR-484

ESCO1	establishment of sister chromatid cohesion N-acetyl-transferase 1	miR-26a
ESR1	estrogen receptor 1	miR-26a; miR-222
ESRRG	estrogen-related receptor gamma	miR-26a
ESYT1	extended synaptotagmin-like protein 1	mir-484
ETF1	eukaryotic translation termination factor 1	miR-26a
ETNK1	ethanolamine kinase 1	miR-26a
ETS1	v-ets avian erythroblastosis virus E26 oncogene homolog 1	miR-222
ETS2	v-ets avian erythroblastosis virus E26 oncogene homolog 2	miR-222
ETV3	ets variant 3	miR-26a; miR-222
ETV6	ets variant 6	mir-484
EVI2B	ecotropic viral integration site 2B	mir-484
EVX2	even-skipped homeobox 2	miR-26a
EXOC5	exocyst complex component 5	mir-484
EXOC8	exocyst complex component 8	miR-26a
EXT1	exostosin glycosyltransferase 1	miR-26a
EXT2	exostosin glycosyltransferase 2	mir-484
EYA3	eyes absent homolog 3 (Drosophila)	miR-26a
EZH1	enhancer of zeste homolog 1 (Drosophila)	mir-484
EZH2	enhancer of zeste homolog 2 (Drosophila)	miR-26a
FA2H	fatty acid 2-hydroxylase	miR-26a
FAF1	Fas (TNFRSF6) associated factor 1	miR-26a
FAIM3	Fas apoptotic inhibitory molecule 3	mir-484
FAM104A	family with sequence similarity 104, member A	mir-484
FAM110A	family with sequence similarity 110, member A	mir-484
FAM118A	family with sequence similarity 118, member A	miR-26a
FAM120A	family with sequence similarity 120A	miR-222; miR-484
FAM120C	family with sequence similarity 120C	mir-484
FAM131B	family with sequence similarity 131, member B	mir-484
FAM136A	family with sequence similarity 136, member A	miR-26a
FAM160B2	family with sequence similarity 160, member B2	mir-484
FAM167A	family with sequence similarity 167, member A	miR-222
FAM168B	family with sequence similarity 168, member B	mir-484
FAM169A	family with sequence similarity 169, member A	miR-26a
FAM178A	family with sequence similarity 178, member A	miR-26a
FAM187A	family with sequence similarity 187, member A	mir-484
FAM189B	family with sequence similarity 189, member B	mir-484
FAM196B	family with sequence similarity 196, member B	miR-26a; miR-222
FAM208A	family with sequence similarity 208, member A	miR-222
FAM214A	family with sequence similarity 214, member A	miR-222
FAM214B	family with sequence similarity 214, member B	miR-26a

FAM46B	family with sequence similarity 46, member B	mir-484
FAM46C	family with sequence similarity 46, member C	miR-26a
FAM49B	family with sequence similarity 49, member B	miR-26a
FAM53A	family with sequence similarity 53, member A	mir-484
FAM53B	family with sequence similarity 53, member B	miR-222
FAM58A	family with sequence similarity 58, member A	mir-484
FAM69C	family with sequence similarity 69, member C	mir-484
FAM83D	family with sequence similarity 83, member D	mir-484
FAM84A	family with sequence similarity 84, member A	mir-484
FAM98A	family with sequence similarity 98, member A	miR-26a
FAM98B	family with sequence similarity 98, member B	miR-26a
FANCA	Fanconi anemia, complementation group A	miR-26a
FAT2	FAT atypical cadherin 2	miR-222
FBN2	fibrillin 2	miR-222
FBRS	fibrosin	mir-484
FBXL19	F-box and leucine-rich repeat protein 19	miR-26a
FBXO11	F-box protein 11	miR-26a; miR-484
FBXO28	F-box protein 28	miR-26a; miR-222
FBXO3	F-box protein 3	miR-222
FBXO42	F-box protein 42	miR-26a
FBXO48	F-box protein 48	miR-26a
FGD1	FYVE, RhoGEF and PH domain containing 1	miR-26a
FGF14	fibroblast growth factor 14	miR-222
FIGN	fidgetin	miR-26a; miR-222
FITM2	fat storage-inducing transmembrane protein 2	mir-484
FKBP3	FK506 binding protein 3, 25kDa	miR-26a
FLT3	fms-related tyrosine kinase 3	mir-484
FMR1	fragile X mental retardation 1	miR-222
FNDC3A	fibronectin type III domain containing 3A	miR-222
FNIP1	folliculin interacting protein 1	miR-26a
FNIP2	folliculin interacting protein 2	miR-222
FOLR2	folate receptor 2 (fetal)	mir-484
FOS	FBJ murine osteosarcoma viral oncogene homolog	miR-222
FOSL2	FOS-like antigen 2	miR-26a
FOXJ1	forkhead box J1	mir-484
FOXK1	forkhead box K1	mir-484
FOXN1	forkhead box N1	miR-26a
FOXN2	forkhead box N2	miR-222
FOXP1	forkhead box P1	miR-222
FOXP2	forkhead box P2	miR-26a; miR-222
FPGT	fucose-1-phosphate guanylyltransferase	mir-484
FRA10AC1	fragile site, folic acid type, rare, fra(10)(q23.3) or fra(10)(q24.2) candidate 1	miR-26a

FRAT2	frequently rearranged in advanced T-cell lymphomas 2	miR-26a
FRK	fyn-related kinase	miR-26a
FRMD4B	FERM domain containing 4B	miR-26a
FRMPD3	FERM and PDZ domain containing 3	mir-484
FRRS1L	ferric-chelate reductase 1-like	miR-222
FRS2	fibroblast growth factor receptor substrate 2	miR-222
FST	follistatin	mir-484
FUBP1	far upstream element (FUSE) binding protein 1	miR-26a
FUT10	fucosyltransferase 10 (alpha (1,3) fucosyltransferase)	mir-484
FUT4	fucosyltransferase 4 (alpha (1,3) fucosyltransferase, myeloid-specific)	mir-484
FUT9	fucosyltransferase 9 (alpha (1,3) fucosyltransferase)	miR-26a; miR-484
G2E3	G2/M-phase specific E3 ubiquitin protein ligase	miR-26a
G3BP2	GTPase activating protein (SH3 domain) binding protein 2	miR-26a
GAB1	GRB2-associated binding protein 1	miR-222
GABRA1	gamma-aminobutyric acid (GABA) A receptor, alpha 1	miR-222
GABRA4	gamma-aminobutyric acid (GABA) A receptor, alpha 4	miR-26a
GAD2	glutamate decarboxylase 2 (pancreatic islets and brain, 65kDa)	miR-26a
GALNT10	UDP-N-acetyl-alpha-D-galactosamine:polypeptide N-acetylgalactosaminyltransferase 10 (GalNAc-T10)	miR-26a; miR-484
GALNT16	UDP-N-acetyl-alpha-D-galactosamine:polypeptide N-acetylgalactosaminyltransferase 16	mir-484
GALNT18	UDP-N-acetyl-alpha-D-galactosamine:polypeptide N-acetylgalactosaminyltransferase 18	miR-222
GALNT7	UDP-N-acetyl-alpha-D-galactosamine:polypeptide N-acetylgalactosaminyltransferase 7 (GalNAc-T7)	miR-26a
GAN	gigaxonin	miR-26a
GAREM	GRB2 associated, regulator of MAPK1	mir-484
GATA4	GATA binding protein 4	miR-26a
GCDH	glutaryl-CoA dehydrogenase	mir-484
GDF11	growth differentiation factor 11	miR-26a; miR-484
GEMIN8	gem (nuclear organelle) associated protein 8	mir-484
GIGYF1	GRB10 interacting GYF protein 1	mir-484
GINS2	GINS complex subunit 2 (Psf2 homolog)	mir-484
GIPC2	GIPC PDZ domain containing family, member 2	mir-484
GIPC3	GIPC PDZ domain containing family, member 3	mir-484
GJA3	gap junction protein, alpha 3, 46kDa	miR-26a
GJA9	gap junction protein, alpha 9, 59kDa	miR-26a
GLCE	glucuronic acid epimerase	mir-484
GLTSCR1L	GLTSCR1-like	miR-26a
GMDS	GDP-mannose 4,6-dehydratase	miR-26a
GMEB1	glucocorticoid modulatory element binding protein 1	miR-26a
GMEB2	glucocorticoid modulatory element binding protein 2	mir-484

GMFB	glia maturation factor, beta	miR-26a
GNA13	guanine nucleotide binding protein (G protein), alpha 13	miR-26a
GNAI2	guanine nucleotide binding protein (G protein), alpha inhibiting activity polypeptide 2	miR-222
GNB2L1	guanine nucleotide binding protein (G protein), beta polypeptide 2-like 1	mir-484
GNE	glucosamine (UDP-N-acetyl)-2-epimerase/N-acetyl-mannosamine kinase	mir-484
GNPNAT1	glucosamine-phosphate N-acetyltransferase 1	miR-26a
GOSR2	golgi SNAP receptor complex member 2	mir-484
GPBP1	GC-rich promoter binding protein 1	miR-222
GPC1	glypican 1	mir-484
GPC4	glypican 4	miR-26a
GPCPD1	glycerophosphocholine phosphodiesterase GDE1 homolog (S. cerevisiae)	mir-484
GPM6A	glycoprotein M6A	miR-222
GPR133	G protein-coupled receptor 133	mir-484
GPR146	G protein-coupled receptor 146	miR-26a
GPR157	G protein-coupled receptor 157	mir-484
GPR161	G protein-coupled receptor 161	miR-26a
GPSM1	G-protein signaling modulator 1	miR-26a
GRB10	growth factor receptor-bound protein 10	miR-26a; miR-484
GRB2	growth factor receptor-bound protein 2	mir-484
GREB1L	growth regulation by estrogen in breast cancer-like	miR-26a; miR-484
GRHL3	grainyhead-like 3 (Drosophila)	miR-26a
GRID2IP	glutamate receptor, ionotropic, delta 2 (Grid2) inter-acting protein	mir-484
GRIN2A	glutamate receptor, ionotropic, N-methyl D-aspartate 2A	miR-26a
GRIN2B	glutamate receptor, ionotropic, N-methyl D-aspartate 2B	miR-26a
GRK7	G protein-coupled receptor kinase 7	mir-484
GRM1	glutamate receptor, metabotropic 1	miR-222
GRSF1	G-rich RNA sequence binding factor 1	miR-26a
GRWD1	glutamate-rich WD repeat containing 1	mir-484
GSK3B	glycogen synthase kinase 3 beta	miR-26a
GSPT1	G1 to S phase transition 1	mir-484
GSTCD	glutathione S-transferase, C-terminal domain contain-ing	mir-484
GTDC1	glycosyltransferase-like domain containing 1	mir-484
GTF3C2	general transcription factor IIIC, polypeptide 2, beta 110kDa	miR-26a
GTF3C5	general transcription factor IIIC, polypeptide 5, 63kDa	mir-484
GTPBP10	GTP-binding protein 10 (putative)	mir-484
GXYLT1	glucoside xylosyltransferase 1	mir-484

GZF1	GDNF-inducible zinc finger protein 1	mir-484
H3F3A	H3 histone, family 3A	miR-222
HAO1	hydroxyacid oxidase (glycolate oxidase) 1	miR-26a
HAPLN1	hyaluronan and proteoglycan link protein 1	miR-26a
HAS2	hyaluronan synthase 2	miR-26a
HAS3	hyaluronan synthase 3	miR-26a
HCN4	hyperpolarization activated cyclic nucleotide-gated potassium channel 4	mir-484
HECTD2	HECT domain containing E3 ubiquitin protein ligase 2	miR-222
HECTD3	HECT domain containing E3 ubiquitin protein ligase 3	miR-26a; miR-484
HECTD4	HECT domain containing E3 ubiquitin protein ligase 4	miR-26a
HEG1	heart development protein with EGF-like domains 1	miR-222
HELZ	helicase with zinc finger	miR-26a
HEPHL1	hephaestin-like 1	miR-26a
HGF	hepatocyte growth factor (hepapoietin A; scatter factor)	miR-26a
HIAT1	hippocampus abundant transcript 1	miR-26a
HIC1	hypermethylated in cancer 1	mir-484
HIC2	hypermethylated in cancer 2	mir-484
HIF1A	hypoxia inducible factor 1, alpha subunit (basic helix-loop-helix transcription factor)	mir-484
HIF1AN	hypoxia inducible factor 1, alpha subunit inhibitor	mir-484
HIPK1	homeodomain interacting protein kinase 1	miR-26a; miR-222
HIPK2	homeodomain interacting protein kinase 2	miR-26a; miR-222
HIPK3	homeodomain interacting protein kinase 3	miR-222
HIRA	histone cell cycle regulator	mir-484
HM13	histocompatibility (minor) 13	mir-484
HMBOX1	homeobox containing 1	miR-26a; miR-222
HMGA1	high mobility group AT-hook 1	miR-26a
HMGA2	high mobility group AT-hook 2	miR-26a
HMGCR	3-hydroxy-3-methylglutaryl-CoA reductase	mir-484
HNF1A	HNF1 homeobox A	mir-484
HNRNPA0	heterogeneous nuclear ribonucleoprotein A0	miR-222
HNRNPA3	heterogeneous nuclear ribonucleoprotein A3	miR-222
HNRNPUL1	heterogeneous nuclear ribonucleoprotein U-like 1	miR-26a
HOMER1	homer homolog 1 (Drosophila)	miR-26a; miR-484
HOOK1	hook microtubule-tethering protein 1	miR-26a
HOXA5	homeobox A5	miR-26a
HOXA7	homeobox A7	miR-222
HOXA9	homeobox A9	miR-26a
HOXD12	homeobox D12	mir-484
HOXD13	homeobox D13	miR-26a
HOXD4	homeobox D4	miR-26a;

		miR-484
HPGD	hydroxyprostaglandin dehydrogenase 15-(NAD)	miR-26a
HRH1	histamine receptor H1	mir-484
HS2ST1	heparan sulfate 2-O-sulfotransferase 1	miR-26a; miR-484
HS6ST1	heparan sulfate 6-O-sulfotransferase 1	miR-26a
HSDL1	hydroxysteroid dehydrogenase like 1	mir-484
HSDL2	hydroxysteroid dehydrogenase like 2	miR-26a
HSH2D	hematopoietic SH2 domain containing	mir-484
HSPA12B	heat shock 70kD protein 12B	mir-484
HSPA13	heat shock protein 70kDa family, member 13	miR-26a
HTR2A	5-hydroxytryptamine (serotonin) receptor 2A, G protein-coupled	miR-26a
HVCN1	hydrogen voltage-gated channel 1	mir-484
HYAL2	hyaluronoglucosaminidase 2	mir-484
ICOS	inducible T-cell co-stimulator	miR-26a; miR-484
IGDCC4	immunoglobulin superfamily, DCC subclass, member 4	mir-484
IGF1	insulin-like growth factor 1 (somatomedin C)	miR-26a
IGF2BP1	insulin-like growth factor 2 mRNA binding protein 1	mir-484
IGF2BP2	insulin-like growth factor 2 mRNA binding protein 2	miR-222
IGFBP3	insulin-like growth factor binding protein 3	mir-484
IGFBP5	insulin-like growth factor binding protein 5	mir-484
IGFBPL1	insulin-like growth factor binding protein-like 1	mir-484
IGLON5	IgLON family member 5	mir-484
IGSF10	immunoglobulin superfamily, member 10	miR-26a
IGSF3	immunoglobulin superfamily, member 3	miR-26a; miR-222; miR-484
IHH	indian hedgehog	mir-484
IKZF2	IKAROS family zinc finger 2 (Helios)	mir-484
IKZF4	IKAROS family zinc finger 4 (Eos)	miR-222
IL10RA	interleukin 10 receptor, alpha	mir-484
IL1RAPL1	interleukin 1 receptor accessory protein-like 1	miR-222
IL20	interleukin 20	miR-26a
IL20RB	interleukin 20 receptor beta	mir-484
IL31	interleukin 31	mir-484
IL6R	interleukin 6 receptor	mir-484
ILF2	interleukin enhancer binding factor 2	mir-484
INHBA	inhibin, beta A	miR-26a
INHBB	inhibin, beta B	miR-26a
INSIG1	insulin induced gene 1	miR-222
INTS2	integrator complex subunit 2	miR-26a
INTU	inturned planar cell polarity protein	miR-26a
IPMK	inositol polyphosphate multikinase	miR-26a
IPO7	importin 7	miR-26a;

		miR-222
IPPK	inositol 1,3,4,5,6-pentakisphosphate 2-kinase	miR-26a
IQCH	IQ motif containing H	mir-484
IQCJ	IQ motif containing J	mir-484
IRF2	interferon regulatory factor 2	miR-222
IRGQ	immunity-related GTPase family, Q	mir-484
IRX5	iroquois homeobox 5	miR-222
ISY1-RAB43	ISY1-RAB43 readthrough	mir-484
ITGA5	integrin, alpha 5 (fibronectin receptor, alpha polypeptide)	miR-26a
ITGB8	integrin, beta 8	miR-26a; miR-222
ITPKB	inositol-trisphosphate 3-kinase B	mir-484
ITPR1	inositol 1,4,5-trisphosphate receptor, type 1	miR-26a
ITPRIPL1	inositol 1,4,5-trisphosphate receptor interacting protein-like 1	miR-26a
JAG1	jagged 1	miR-26a
JAKMIP2	janus kinase and microtubule interacting protein 2	miR-26a
JAM3	junctional adhesion molecule 3	miR-26a
JARID2	jumonji, AT rich interactive domain 2	miR-26a
JPH4	junctophilin 4	mir-484
KALRN	kalirin, RhoGEF kinase	mir-484
KANSL1	KAT8 regulatory NSL complex subunit 1	miR-222
KAZN	kazrin, periplakin interacting protein	miR-26a; miR-484
KBTBD13	kelch repeat and BTB (POZ) domain containing 13	mir-484
KBTBD8	kelch repeat and BTB (POZ) domain containing 8	miR-26a; miR-484
KCMF1	potassium channel modulatory factor 1	miR-26a
KCNA5	potassium voltage-gated channel, shaker-related subfamily, member 5	mir-484
KCNC3	potassium voltage-gated channel, Shaw-related subfamily, member 3	mir-484
KCNE4	potassium voltage-gated channel, Isk-related family, member 4	miR-26a
KCNH7	potassium voltage-gated channel, subfamily H (eag-related), member 7	miR-26a
KCNJ2	potassium inwardly-rectifying channel, subfamily J, member 2	miR-26a
KCNK1	potassium channel, subfamily K, member 1	miR-26a
KCNK13	potassium channel, subfamily K, member 13	mir-484
KCNN3	potassium intermediate/small conductance calcium-activated channel, subfamily N, member 3	miR-26a
KCNQ3	potassium voltage-gated channel, KQT-like subfamily, member 3	miR-222
KCNQ4	potassium voltage-gated channel, KQT-like subfamily, member 4	miR-26a
KCTD10	potassium channel tetramerization domain containing 10	mir-484

KCTD18	potassium channel tetramerization domain containing 18	miR-26a
KCTD3	potassium channel tetramerization domain containing 3	mir-484
KDM4A	lysine (K)-specific demethylase 4A	mir-484
KDM5B	lysine (K)-specific demethylase 5B	mir-484
KDM6A	lysine (K)-specific demethylase 6A	miR-26a
KDR	kinase insert domain receptor (a type III receptor tyrosine kinase)	miR-222
KHDRBS2	KH domain containing, RNA binding, signal transduction associated 2	miR-222
KIF16B	kinesin family member 16B	miR-222
KIF21B	kinesin family member 21B	miR-26a
KIF2A	kinesin heavy chain member 2A	mir-484
KIF6	kinesin family member 6	mir-484
KIT	v-kit Hardy-Zuckerman 4 feline sarcoma viral oncogene homolog	miR-222
KLF10	Kruppel-like factor 10	miR-26a
KLF4	Kruppel-like factor 4 (gut)	miR-26a
KLF7	Kruppel-like factor 7 (ubiquitous)	miR-26a; miR-222
KLHL15	kelch-like family member 15	miR-26a
KLHL18	kelch-like family member 18	miR-26a
KLHL2	kelch-like family member 2	mir-484
KLHL22	kelch-like family member 22	mir-484
KLHL42	kelch-like family member 42	miR-26a
KMT2A	lysine (K)-specific methyltransferase 2A	miR-222; miR-484
KMT2C	lysine (K)-specific methyltransferase 2C	miR-26a; miR-222
KNDC1	kinase non-catalytic C-lobe domain (KIND) containing 1	mir-484
KPNA2	karyopherin alpha 2 (RAG cohort 1, importin alpha 1)	miR-26a; miR-222
KPNA3	karyopherin alpha 3 (importin alpha 4)	miR-26a
KPNA5	karyopherin alpha 5 (importin alpha 6)	miR-26a
KPNA6	karyopherin alpha 6 (importin alpha 7)	miR-26a
KRBA1	KRAB-A domain containing 1	mir-484
KRT1	keratin 1	mir-484
KRT222	keratin 222	miR-26a
KRTCAP3	keratinocyte associated protein 3	mir-484
KSR1	kinase suppressor of ras 1	miR-222
LAMC3	laminin, gamma 3	mir-484
LARP1	La ribonucleoprotein domain family, member 1	miR-26a
LARP4	La ribonucleoprotein domain family, member 4	miR-26a
LARP4B	La ribonucleoprotein domain family, member 4B	miR-26a
LCN6	lipocalin 6	mir-484
LDLR	low density lipoprotein receptor	mir-484

LEF1	lymphoid enhancer-binding factor 1	miR-26a
LETM1	leucine zipper-EF-hand containing transmembrane protein 1	mir-484
LGI2	leucine-rich repeat LGI family, member 2	mir-484
LHFP	lipoma HMGIC fusion partner	mir-484
LHFPL2	lipoma HMGIC fusion partner-like 2	miR-222
LHFPL4	lipoma HMGIC fusion partner-like 4	miR-26a
LHX1	LIM homeobox 1	miR-26a
LHX8	LIM homeobox 8	miR-222
LIF	leukemia inhibitory factor	mir-484
LIFR	leukemia inhibitory factor receptor alpha	miR-222
LIG3	ligase III, DNA, ATP-dependent	mir-484
LIMK2	LIM domain kinase 2	mir-484
LIN52	lin-52 homolog (C. elegans)	miR-26a
LIN54	lin-54 homolog (C. elegans)	mir-484
LINGO1	leucine rich repeat and Ig domain containing 1	miR-26a
LMAN1	lectin, mannose-binding, 1	miR-26a
LMLN	leishmanolysin-like (metallopeptidase M8 family)	miR-26a
LMX1A	LIM homeobox transcription factor 1, alpha	miR-222
LNPEP	leucyl/cystinyl aminopeptidase	miR-26a
LNX2	ligand of numb-protein X 2	miR-26a
LONRF3	LON peptidase N-terminal domain and ring finger 3	mir-484
LOX	lysyl oxidase	miR-26a
LOXL2	lysyl oxidase-like 2	miR-26a
LPHN1	latrophilin 1	miR-26a
LPP	LIM domain containing preferred translocation partner in lipoma	miR-26a
LPPR1	Lipid phosphate phosphatase-related protein type 1	miR-222
LRCH1	leucine-rich repeats and calponin homology (CH) domain containing 1	miR-26a
LRFN2	leucine rich repeat and fibronectin type III domain containing 2	miR-222
LRRC14	leucine rich repeat containing 14	miR-222
LRRC15	leucine rich repeat containing 15	mir-484
LRRC16A	leucine rich repeat containing 16A	miR-26a
LRRC3	leucine rich repeat containing 3	mir-484
LRRC41	leucine rich repeat containing 41	mir-484
LRRC56	leucine rich repeat containing 56	mir-484
LRRC59	leucine rich repeat containing 59	miR-26a
LRRFIP1	leucine rich repeat (in FLII) interacting protein 1	mir-484
LSM11	LSM11, U7 small nuclear RNA associated	miR-26a
LSM12	LSM12 homolog (S. cerevisiae)	miR-26a
LTBP1	latent transforming growth factor beta binding protein 1	miR-26a
LUC7L3	LUC7-like 3 (S. cerevisiae)	miR-222
LUZP1	leucine zipper protein 1	mir-484
LUZP6	leucine zipper protein 6	miR-26a

LY6D	lymphocyte antigen 6 complex, locus D	mir-484
LYPLA2	lysophospholipase II	mir-484
LYRM9	LYR motif containing 9	mir-484
LYSMD3	LysM, putative peptidoglycan-binding, domain containing 3	mir-484
LZIC	leucine zipper and CTNNBIP1 domain containing	mir-484
MAB21L1	mab-21-like 1 (C. elegans)	miR-26a
MAFK	v-maf avian musculoaponeurotic fibrosarcoma oncogene homolog K	mir-484
MAGI1	membrane associated guanylate kinase, WW and PDZ domain containing 1	miR-222
MAGI2	membrane associated guanylate kinase, WW and PDZ domain containing 2	miR-222
MAGI3	membrane associated guanylate kinase, WW and PDZ domain containing 3	miR-26a
MAL2	mal, T-cell differentiation protein 2 (gene/pseudogene)	miR-26a
MAML1	mastermind-like 1 (Drosophila)	mir-484
MAN1A2	mannosidase, alpha, class 1A, member 2	miR-26a
MAN2A1	mannosidase, alpha, class 2A, member 1	miR-26a
MANBA	mannosidase, beta A, lysosomal	mir-484
MANEAL	mannosidase, endo-alpha-like	mir-484
MANF	mesencephalic astrocyte-derived neurotrophic factor	mir-484
MAP1A	microtubule-associated protein 1A	miR-26a
MAP1B	microtubule-associated protein 1B	miR-26a
MAP2	microtubule-associated protein 2	miR-26a
MAP3K1	mitogen-activated protein kinase kinase kinase 1, E3 ubiquitin protein ligase	miR-26a
MAP3K11	mitogen-activated protein kinase kinase kinase 11	mir-484
MAP3K2	mitogen-activated protein kinase kinase kinase 2	miR-26a
MAP3K9	mitogen-activated protein kinase kinase kinase 9	miR-26a
MAP7	microtubule-associated protein 7	miR-26a
MAP9	microtubule-associated protein 9	miR-26a
MAPK10	mitogen-activated protein kinase 10	miR-222
MAPK6	mitogen-activated protein kinase 6	miR-26a; miR-222
MAPK9	mitogen-activated protein kinase 9	mir-484
MAPKAPK2	mitogen-activated protein kinase-activated protein kinase 2	mir-484
MARCH5	membrane-associated ring finger (C3HC4) 5	mir-484
MARK1	MAP/microtubule affinity-regulating kinase 1	miR-26a; miR-222
MAT2A	methionine adenosyltransferase II, alpha	miR-26a
MATR3	matrin 3	miR-26a
MB	myoglobin	mir-484
MBD2	methyl-CpG binding domain protein 2	miR-222
MBTD1	mbt domain containing 1	mir-484
MCC	mutated in colorectal cancers	miR-26a
MCL1	myeloid cell leukemia sequence 1 (BCL2-related)	miR-26a

MCM2	minichromosome maintenance complex component 2	mir-484
MDN1	MDN1, midasin homolog (yeast)	miR-26a
MED13L	mediator complex subunit 13-like	miR-26a
MED6	mediator complex subunit 6	mir-484
MEF2C	myocyte enhancer factor 2C	miR-26a
MEGF9	multiple EGF-like-domains 9	miR-26a; miR-222
MEIS1	Meis homeobox 1	miR-222
MESDC1	mesoderm development candidate 1	miR-222
METTL2A	methyltransferase like 2A	mir-484
MEX3A	mex-3 RNA binding family member A	miR-222
MEX3B	mex-3 RNA binding family member B	miR-26a; miR-484
MFAP2	microfibrillar-associated protein 2	mir-484
MFAP3	microfibrillar-associated protein 3	mir-484
MFAP3L	microfibrillar-associated protein 3-like	miR-26a
MFHAS1	malignant fibrous histiocytoma amplified sequence 1	miR-26a
MFN1	mitofusin 1	miR-26a; miR-222
MFN2	mitofusin 2	mir-484
MFSD10	major facilitator superfamily domain containing 10	mir-484
MFSD6	major facilitator superfamily domain containing 6	miR-26a
MGAT4A	mannosyl (alpha-1,3-)-glycoprotein beta-1,4-N-acetyl-glucosaminyltransferase, isozyme A	miR-26a
MGAT5	mannosyl (alpha-1,6-)-glycoprotein beta-1,6-N-acetyl-glucosaminyltransferase	mir-484
MGRN1	mahogunin ring finger 1, E3 ubiquitin protein ligase	mir-484
MGST2	microsomal glutathione S-transferase 2	mir-484
MIA3	melanoma inhibitory activity family, member 3	miR-222
MIB1	mindbomb E3 ubiquitin protein ligase 1	miR-26a
MICAL3	microtubule associated monooxygenase, calponin and LIM domain containing 3	miR-26a
MICU3	mitochondrial calcium uptake family, member 3	mir-484
MID1	midline 1 (Opitz/BBB syndrome)	miR-222
MIDN	midnolin	miR-222
MIER3	mesoderm induction early response 1, family member 3	miR-26a; miR-222
MINK1	misshapen-like kinase 1	mir-484
MITF	microphthalmia-associated transcription factor	miR-26a
MKNK2	MAP kinase interacting serine/threonine kinase 2	miR-26a
MLEC	malectin	miR-26a
MLLT3	myeloid/lymphoid or mixed-lineage leukemia (trithorax homolog, Drosophila); translocated to, 3	miR-26a
MLLT6	myeloid/lymphoid or mixed-lineage leukemia (trithorax homolog, Drosophila); translocated to, 6	miR-222; miR-484
MLXIP	MLX interacting protein	mir-484
MMP14	matrix metallopeptidase 14 (membrane-inserted)	miR-26a
MMP16	matrix metallopeptidase 16 (membrane-inserted)	miR-26a

MN1	meningioma (disrupted in balanced translocation) 1	mir-484
MNX1	motor neuron and pancreas homeobox 1	miR-26a
MOB1A	MOB kinase activator 1A	miR-26a
MOB3C	MOB kinase activator 3C	mir-484
MOGAT2	monoacylglycerol O-acyltransferase 2	mir-484
MON2	MON2 homolog (S. cerevisiae)	miR-222
MPP6	membrane protein, palmitoylated 6 (MAGUK p55 subfamily member 6)	miR-26a
MPRIP	myosin phosphatase Rho interacting protein	miR-26a
MRAS	muscle RAS oncogene homolog	miR-26a
MREG	melanoregulin	miR-26a
MRPL27	mitochondrial ribosomal protein L27	mir-484
MS4A6A	membrane-spanning 4-domains, subfamily A, member 6A	mir-484
MSANTD1	Myb/SANT-like DNA-binding domain containing 1	miR-26a
MSANTD3	Myb/SANT-like DNA-binding domain containing 3	mir-484
MSI2	musashi RNA-binding protein 2	mir-484
MSL1	male-specific lethal 1 homolog (Drosophila)	miR-222
MSL2	male-specific lethal 2 homolog (Drosophila)	miR-222
MST4	Serine/threonine-protein kinase MST4	miR-222
MTCH1	mitochondrial carrier 1	mir-484
MTDH	metadherin	miR-26a
MTG1	mitochondrial ribosome-associated GTPase 1	mir-484
MTMR11	myotubularin related protein 11	mir-484
MTPN	myotrophin	miR-26a
MTSS1L	metastasis suppressor 1-like	mir-484
MTX2	metaxin 2	miR-26a
MXI1	MAX interactor 1, dimerization protein	miR-26a
MYCBP	MYC binding protein	miR-26a
MYLIP	myosin regulatory light chain interacting protein	miR-222
MYO10	myosin X	miR-222
MYO1A	myosin IA	mir-484
MYO1D	myosin ID	mir-484
MYO9A	myosin IXA	miR-26a
MYOM3	myomesin 3	mir-484
MYOZ3	myozenin 3	mir-484
MYPOP	Myb-related transcription factor, partner of profilin	miR-26a
MYT1L	myelin transcription factor 1-like	miR-222
MZB1	marginal zone B and B1 cell-specific protein	mir-484
NAA15	N(alpha)-acetyltransferase 15, NatA auxiliary subunit	miR-26a
NAA25	N(alpha)-acetyltransferase 25, NatB auxiliary subunit	miR-222
NAA40	N(alpha)-acetyltransferase 40, NatD catalytic subunit	mir-484
NAB1	NGFI-A binding protein 1 (EGR1 binding protein 1)	miR-26a
NABP1	nucleic acid binding protein 1	miR-26a
NACC1	nucleus accumbens associated 1, BEN and BTB (POZ) domain containing	mir-484

NACC2	NACC family member 2, BEN and BTB (POZ) domain containing	miR-26a
NAGPA	N-acetylglucosamine-1-phosphodiester alpha-N-acetylglucosaminidase	miR-26a
NAMPT	nicotinamide phosphoribosyltransferase	miR-26a
NANOS1	nanos homolog 1 (Drosophila)	miR-222
NAP1L1	nucleosome assembly protein 1-like 1	miR-222
NAP1L5	nucleosome assembly protein 1-like 5	miR-26a; miR-222
NAT9	N-acetyltransferase 9 (GCN5-related, putative)	mir-484
NCAM2	neural cell adhesion molecule 2	miR-26a
NCEH1	neutral cholesterol ester hydrolase 1	miR-26a
NCS1	neuronal calcium sensor 1	mir-484
NDFIP1	Nedd4 family interacting protein 1	miR-222
NDFIP2	Nedd4 family interacting protein 2	miR-26a
NEBL	nebulette	miR-26a
NEDD4L	neural precursor cell expressed, developmentally down-regulated 4-like, E3 ubiquitin protein ligase	mir-484
NEK10	NIMA-related kinase 10	miR-26a
NEK6	NIMA-related kinase 6	miR-26a
NETO1	neuropilin (NRP) and tolloid (TLL)-like 1	miR-26a
NFAT5	nuclear factor of activated T-cells 5, tonicity-responsive	miR-222
NFATC2IP	nuclear factor of activated T-cells, cytoplasmic, calcineurin-dependent 2 interacting protein	mir-484
NFATC3	nuclear factor of activated T-cells, cytoplasmic, calcineurin-dependent 3	miR-222
NFATC4	nuclear factor of activated T-cells, cytoplasmic, calcineurin-dependent 4	mir-484
NFE2L3	nuclear factor, erythroid 2-like 3	miR-26a
NFIB	nuclear factor I/B	mir-484
NHS	Nance-Horan syndrome (congenital cataracts and dental anomalies)	miR-26a
NHSL1	NHS-like 1	miR-26a
NIPAL4	NIPA-like domain containing 4	miR-222
NIPBL	Nipped-B homolog (Drosophila)	miR-26a; miR-222
NKRF	NFKB repressing factor	miR-26a
NKX2-5	NK2 homeobox 5	miR-26a
NLK	nemo-like kinase	miR-26a; miR-222
NMRK1	nicotinamide riboside kinase 1	mir-484
NOL8	nucleolar protein 8	mir-484
NOS1AP	nitric oxide synthase 1 (neuronal) adaptor protein	mir-484
NOTCH2NL	notch 2 N-terminal like	mir-484
NOVA1	neuro-oncological ventral antigen 1	miR-26a; miR-222
NR0B2	nuclear receptor subfamily 0, group B, member 2	mir-484

NR4A1	nuclear receptor subfamily 4, group A, member 1	miR-222
NR4A3	nuclear receptor subfamily 4, group A, member 3	mir-484
NRAS	neuroblastoma RAS viral (v-ras) oncogene homolog	miR-26a
NRG1	neuregulin 1	miR-222
NRG2	neuregulin 2	miR-26a
NRIP1	nuclear receptor interacting protein 1	miR-26a
NRK	Nik related kinase	miR-222
NSG1	Neuron-specific protein family member 1	mir-484
NSMCE4A	non-SMC element 4 homolog A (S. cerevisiae)	miR-222
NSUN3	NOP2/Sun domain family, member 3	miR-26a
NSUN4	NOP2/Sun domain family, member 4	mir-484
NTF3	neurotrophin 3	miR-222
NTPCR	nucleoside-triphosphatase, cancer-related	mir-484
NTRK3	neurotrophic tyrosine kinase, receptor, type 3	mir-484
NUDT3	nudix (nucleoside diphosphate linked moiety X)-type motif 3	mir-484
NUFIP2	nuclear fragile X mental retardation protein interacting protein 2	miR-222
NUP153	nucleoporin 153kDa	miR-26a
NUP50	nucleoporin 50kDa	miR-26a
NUP98	nucleoporin 98kDa	mir-484
NUS1	nuclear undecaprenyl pyrophosphate synthase 1 homolog (S. cerevisiae)	miR-26a
NXN	nucleoredoxin	mir-484
NXPH1	neurexophilin 1	miR-222
OAF	OAF homolog (Drosophila)	miR-26a; miR-484
OARD1	O-acyl-ADP-ribose deacylase 1	mir-484
OBFC1	oligonucleotide/oligosaccharide-binding fold containing 1	miR-26a
OCLN	occludin	miR-26a
OGG1	8-oxoguanine DNA glycosylase	mir-484
ONECUT3	one cut homeobox 3	miR-26a
OPA3	optic atrophy 3 (autosomal recessive, with chorea and spastic paraplegia)	mir-484
OS9	osteosarcoma amplified 9, endoplasmic reticulum lectin	mir-484
OSBPL11	oxysterol binding protein-like 11	miR-26a
OSBPL2	oxysterol binding protein-like 2	miR-26a
OSR1	odd-skipped related 1 (Drosophila)	mir-484
OTUD1	OTU domain containing 1	miR-26a
OTUD4	OTU domain containing 4	miR-26a
OXTR	oxytocin receptor	mir-484
P2RX6	purinergic receptor P2X, ligand-gated ion channel, 6	mir-484
P2RY2	purinergic receptor P2Y, G-protein coupled, 2	mir-484
PAFAH1B1	platelet-activating factor acetylhydrolase 1b, regulatory subunit 1 (45kDa)	miR-222

PAFAH1B2	platelet-activating factor acetylhydrolase 1b, catalytic subunit 2 (30kDa)	mir-484
PAIP1	poly(A) binding protein interacting protein 1	miR-222
PAIP2	poly(A) binding protein interacting protein 2	miR-222
PAK1	p21 protein (Cdc42/Rac)-activated kinase 1	miR-222
PAK2	p21 protein (Cdc42/Rac)-activated kinase 2	miR-26a
PALM	paralemmin	mir-484
PALM2	paralemmin 2	mir-484
PALM3	paralemmin 3	miR-26a
PAN3	PAN3 poly(A) specific ribonuclease subunit homolog (S. cerevisiae)	miR-26a
PAPD4	PAP associated domain containing 4	miR-26a
PARP1	poly (ADP-ribose) polymerase 1	mir-484
PARP11	poly (ADP-ribose) polymerase family, member 11	mir-484
PATL1	protein associated with topoisomerase II homolog 1 (yeast)	miR-26a
PATZ1	POZ (BTB) and AT hook containing zinc finger 1	miR-26a
PAWR	PRKC, apoptosis, WT1, regulator	miR-26a
PAX5	paired box 5	miR-26a
PAX9	paired box 9	mir-484
PBX3	pre-B-cell leukemia homeobox 3	miR-222
PCDH18	protocadherin 18	miR-26a
PCDH19	protocadherin 19	mir-484
PCDHA1	protocadherin alpha 1	miR-222
PCDHA10	protocadherin alpha 10	miR-222
PCDHA11	protocadherin alpha 11	miR-222
PCDHA12	protocadherin alpha 12	miR-222
PCDHA13	protocadherin alpha 13	miR-222
PCDHA2	protocadherin alpha 2	miR-222
PCDHA3	protocadherin alpha 3	miR-222
PCDHA4	protocadherin alpha 4	miR-222
PCDHA5	protocadherin alpha 5	miR-222
PCDHA6	protocadherin alpha 6	miR-222
PCDHA7	protocadherin alpha 7	miR-222
PCDHA8	protocadherin alpha 8	miR-222
PCDHA9	protocadherin alpha 9	miR-222
PCDHAC1	protocadherin alpha subfamily C, 1	miR-222
PCDHAC2	protocadherin alpha subfamily C, 2	miR-222
PCNX	pecanex homolog (Drosophila)	miR-26a
PDAP1	PDGFA associated protein 1	mir-484
PDCD10	programmed cell death 10	miR-26a; miR-222
PDCD6IP	programmed cell death 6 interacting protein	mir-484
PDE3A	phosphodiesterase 3A, cGMP-inhibited	miR-222
PDE4B	phosphodiesterase 4B, cAMP-specific	miR-26a
PDE4D	phosphodiesterase 4D, cAMP-specific	miR-26a
PDE7A	phosphodiesterase 7A	miR-26a

PDGFA	platelet-derived growth factor alpha polypeptide	miR-222
PDHX	pyruvate dehydrogenase complex, component X	miR-26a
PDK2	pyruvate dehydrogenase kinase, isozyme 2	mir-484
PDK3	pyruvate dehydrogenase kinase, isozyme 3	mir-484
PDS5A	PDS5, regulator of cohesion maintenance, homolog A (S. cerevisiae)	mir-484
PDX1	pancreatic and duodenal homeobox 1	mir-484
PDXK	pyridoxal (pyridoxine, vitamin B6) kinase	mir-484
PDZRN4	PDZ domain containing ring finger 4	miR-222
PELI2	pellino E3 ubiquitin protein ligase family member 2	miR-26a
PEX11B	peroxisomal biogenesis factor 11 beta	mir-484
PEX6	peroxisomal biogenesis factor 6	mir-484
PEX7	peroxisomal biogenesis factor 7	mir-484
PFDN4	prefoldin subunit 4	miR-26a
PFKFB2	6-phosphofructo-2-kinase/fructose-2,6-biphosphatase 2	miR-26a; miR-484
PFKFB3	6-phosphofructo-2-kinase/fructose-2,6-biphosphatase 3	miR-26a
PFKL	phosphofructokinase, liver	mir-484
PGAP1	post-GPI attachment to proteins 1	miR-222
PGBD5	piggyBac transposable element derived 5	miR-222
PGD	phosphogluconate dehydrogenase	mir-484
PGF	placental growth factor	mir-484
PGM2L1	phosphoglucomutase 2-like 1	miR-26a
PGR	progesterone receptor	miR-26a
PGRMC2	progesterone receptor membrane component 2	miR-26a
PHACTR1	phosphatase and actin regulator 1	mir-484
PHACTR4	phosphatase and actin regulator 4	miR-222
PHF12	PHD finger protein 12	mir-484
PHF2	PHD finger protein 2	miR-222
PHF20L1	PHD finger protein 20-like 1	miR-26a
PHF21A	PHD finger protein 21A	miR-26a
PHF3	PHD finger protein 3	miR-26a
PHF6	PHD finger protein 6	miR-26a
PHLDB2	pleckstrin homology-like domain, family B, member 2	miR-26a
PHOX2B	paired-like homeobox 2b	mir-484
PHRF1	PHD and ring finger domains 1	mir-484
PIANP	PILR alpha associated neural protein	mir-484
PIAS1	protein inhibitor of activated STAT, 1	miR-26a
PIEZO2	piezo-type mechanosensitive ion channel component 2	miR-222
PIGG	phosphatidylinositol glycan anchor biosynthesis, class G	miR-26a; miR-484
PIGO	phosphatidylinositol glycan anchor biosynthesis, class O	mir-484
PIGU	phosphatidylinositol glycan anchor biosynthesis, class U	miR-26a

PIK3C2A	phosphatidylinositol-4-phosphate 3-kinase, catalytic subunit type 2 alpha	miR-26a
PIK3CB	phosphatidylinositol-4,5-bisphosphate 3-kinase, catalytic subunit beta	miR-26a
PIK3CD	phosphatidylinositol-4,5-bisphosphate 3-kinase, catalytic subunit delta	mir-484
PIK3R1	phosphoinositide-3-kinase, regulatory subunit 1 (alpha)	miR-222; miR-484
PIK3R3	phosphoinositide-3-kinase, regulatory subunit 3 (gamma)	miR-26a
PIKFYVE	phosphoinositide kinase, FYVE finger containing	mir-484
PIM3	pim-3 oncogene	miR-26a
PIP5K1B	phosphatidylinositol-4-phosphate 5-kinase, type I, beta	mir-484
PITPNC1	phosphatidylinositol transfer protein, cytoplasmic 1	miR-26a
PITPNM2	phosphatidylinositol transfer protein, membrane-associated 2	miR-222
PKDCC	protein kinase domain containing, cytoplasmic	miR-222
PKIA	protein kinase (cAMP-dependent, catalytic) inhibitor alpha	miR-222; miR-484
PLA2G4E	phospholipase A2, group IVE	mir-484
PLCB1	phospholipase C, beta 1 (phosphoinositide-specific)	miR-26a
PLCB2	phospholipase C, beta 2	mir-484
PLCB3	phospholipase C, beta 3 (phosphatidylinositol-specific)	mir-484
PLCD3	phospholipase C, delta 3	mir-484
PLEKHA2	pleckstrin homology domain containing, family A (phosphoinositide binding specific) member 2	miR-222
PLEKHB2	pleckstrin homology domain containing, family B (evectins) member 2	mir-484
PLEKHH1	pleckstrin homology domain containing, family H (with MyTH4 domain) member 1	miR-26a
PLOD2	procollagen-lysine, 2-oxoglutarate 5-dioxygenase 2	miR-26a
PLXNA2	plexin A2	miR-26a
PLXNC1	plexin C1	miR-222
PMEPA1	prostate transmembrane protein, androgen induced 1	miR-26a
PNO1	partner of NOB1 homolog (S. cerevisiae)	mir-484
PNPLA3	patatin-like phospholipase domain containing 3	mir-484
PNRC1	proline-rich nuclear receptor coactivator 1	miR-26a
PODNL1	podocan-like 1	mir-484
POGZ	pogo transposable element with ZNF domain	miR-222
POM121C	POM121 transmembrane nucleoporin C	miR-26a
POMGNT1	protein O-linked mannose N-acetylglucosaminyltransferase 1 (beta 1,2-)	mir-484
POP4	processing of precursor 4, ribonuclease P/MRP subunit (S. cerevisiae)	mir-484
POU4F1	POU class 4 homeobox 1	miR-26a
POU6F2	POU class 6 homeobox 2	mir-484

PPARGC1B	peroxisome proliferator-activated receptor gamma, co-activator 1 beta	miR-26a
PPEF1	protein phosphatase, EF-hand calcium binding domain 1	mir-484
PPIG	peptidylprolyl isomerase G (cyclophilin G)	miR-26a
PPP1R15B	protein phosphatase 1, regulatory subunit 15B	miR-26a; miR-222
PPP2R2A	protein phosphatase 2, regulatory subunit B, alpha	miR-222
PPP3CB	protein phosphatase 3, catalytic subunit, beta isozyme	miR-26a
PPP3R1	protein phosphatase 3, regulatory subunit B, alpha	miR-26a; miR-222
PPP4R1	protein phosphatase 4, regulatory subunit 1	miR-26a
PPP6C	protein phosphatase 6, catalytic subunit	miR-26a; miR-222
PRDM11	PR domain containing 11	mir-484
PRDM2	PR domain containing 2, with ZNF domain	mir-484
PRELP	proline/arginine-rich end leucine-rich repeat protein	miR-26a
PRKAA2	protein kinase, AMP-activated, alpha 2 catalytic subunit	miR-26a
PRKACB	protein kinase, cAMP-dependent, catalytic, beta	miR-222
PRKAG2	protein kinase, AMP-activated, gamma 2 non-catalytic subunit	miR-26a
PRKAG3	protein kinase, AMP-activated, gamma 3 non-catalytic subunit	mir-484
PRKCD	protein kinase C, delta	miR-26a
PRKCQ	protein kinase C, theta	miR-26a
PRKG1	protein kinase, cGMP-dependent, type I	miR-26a
PRM1	protamine 1	mir-484
PROM2	prominin 2	mir-484
PRR14L	proline rich 14-like	miR-222
PRR5L	proline rich 5 like	miR-26a
PRRC2B	proline-rich coiled-coil 2B	mir-484
PRRG3	proline rich Gla (G-carboxyglutamic acid) 3 (transmembrane)	miR-222
PRUNE	prune exopolyphosphatase	miR-222
PSD3	pleckstrin and Sec7 domain containing 3	miR-26a
PSMB5	proteasome (prosome, macropain) subunit, beta type, 5	miR-222
PSMC6	proteasome (prosome, macropain) 26S subunit, ATPase, 6	mir-484
PSMF1	proteasome (prosome, macropain) inhibitor subunit 1 (PI31)	mir-484
PTBP2	polypyrimidine tract binding protein 2	miR-222
PTBP3	polypyrimidine tract binding protein 3	miR-222
PTCHD1	patched domain containing 1	miR-26a; miR-222
PTEN	phosphatase and tensin homolog	miR-26a
PTGIS	prostaglandin I2 (prostacyclin) synthase	mir-484

PTGS2	prostaglandin-endoperoxide synthase 2 (prostaglandin G/H synthase and cyclooxygenase)	miR-26a
PTK7	protein tyrosine kinase 7	mir-484
PTP4A1	protein tyrosine phosphatase type IVA, member 1	miR-26a
PTPLA	protein tyrosine phosphatase-like (proline instead of catalytic arginine), member A	miR-26a
PTPN13	protein tyrosine phosphatase, non-receptor type 13 (APO-1/CD95 (Fas)-associated phosphatase)	miR-26a
PTPN3	protein tyrosine phosphatase, non-receptor type 3	miR-26a
PTPRD	protein tyrosine phosphatase, receptor type, D	miR-26a
PTS	6-pyruvoyltetrahydropterin synthase	miR-222
PURA	purine-rich element binding protein A	miR-26a; miR-222
PVRL1	poliovirus receptor-related 1 (herpesvirus entry mediator C)	miR-222
PWWP2A	PWWP domain containing 2A	miR-26a
PYGO2	pygopus homolog 2 (Drosophila)	mir-484
QKI	QKI, KH domain containing, RNA binding	miR-222
R3HCC1L	R3H domain and coiled-coil containing 1-like	mir-484
RAB11A	RAB11A, member RAS oncogene family	miR-26a
RAB11FIP1	RAB11 family interacting protein 1 (class I)	miR-26a; miR-484
RAB11FIP4	RAB11 family interacting protein 4 (class II)	mir-484
RAB11FIP5	RAB11 family interacting protein 5 (class I)	mir-484
RAB1A	RAB1A, member RAS oncogene family	miR-222
RAB31	RAB31, member RAS oncogene family	mir-484
RAB37	RAB37, member RAS oncogene family	mir-484
RAB3B	RAB3B, member RAS oncogene family	miR-222
RAB3GAP2	RAB3 GTPase activating protein subunit 2 (non-catalytic)	miR-222
RAB43	RAB43, member RAS oncogene family	mir-484
RABIF	RAB interacting factor	mir-484
RALGAPA1	Ral GTPase activating protein, alpha subunit 1 (catalytic)	miR-222
RANBP10	RAN binding protein 10	miR-26a
RANBP2	RAN binding protein 2	miR-222
RANBP9	RAN binding protein 9	miR-26a
RAP1A	RAP1A, member of RAS oncogene family	miR-26a; miR-484
RAP1B	RAP1B, member of RAS oncogene family	miR-26a
RAP2C	RAP2C, member of RAS oncogene family	miR-26a
RASA4	RAS p21 protein activator 4	mir-484
RASAL2	RAS protein activator like 2	miR-26a
RASSF3	Ras association (RalGDS/AF-6) domain family member 3	miR-26a
RASSF5	Ras association (RalGDS/AF-6) domain family member 5	mir-484
RB1	retinoblastoma 1	miR-26a

RB1CC1	RB1-inducible coiled-coil 1	mir-484
RBM17	RNA binding motif protein 17	mir-484
RBM24	RNA binding motif protein 24	miR-26a; miR-222
RBM47	RNA binding motif protein 47	miR-26a
RBMS1	RNA binding motif, single stranded interacting protein 1	miR-26a
RBP4	retinol binding protein 4, plasma	mir-484
RCBTB1	regulator of chromosome condensation (RCC1) and BTB (POZ) domain containing protein 1	miR-26a
RCN1	reticulocalbin 1, EF-hand calcium binding domain	miR-26a
RCN2	reticulocalbin 2, EF-hand calcium binding domain	miR-26a
RCOR1	REST corepressor 1	miR-26a
RDH14	retinol dehydrogenase 14 (all-trans/9-cis/11-cis)	miR-26a
REEP3	receptor accessory protein 3	miR-26a
REEP4	receptor accessory protein 4	miR-26a
RERE	arginine-glutamic acid dipeptide (RE) repeats	miR-26a
REST	RE1-silencing transcription factor	miR-26a
REV1	REV1, polymerase (DNA directed)	miR-222
REV3L	REV3-like, polymerase (DNA directed), zeta, catalytic subunit	miR-222
RFX3	regulatory factor X, 3 (influences HLA class II expression)	miR-26a; miR-222; miR-484
RFX7	regulatory factor X, 7	miR-26a; miR-222
RGS17	regulator of G-protein signaling 17	miR-26a; miR-222; miR-484
RGS4	regulator of G-protein signaling 4	miR-26a
RGS9BP	regulator of G protein signaling 9 binding protein	mir-484
RHOQ	ras homolog family member Q	miR-26a; miR-484
RHPN2	rhophilin, Rho GTPase binding protein 2	mir-484
RIMKLA	ribosomal modification protein rimK-like family member A	miR-26a
RIMS3	regulating synaptic membrane exocytosis 3	miR-222; miR-484
RIOK3	RIO kinase 3	mir-484
RLF	rearranged L-myc fusion	miR-26a
RLIM	ring finger protein, LIM domain interacting	mir-484
RNF150	ring finger protein 150	miR-26a
RNF213	ring finger protein 213	miR-26a
RNF214	ring finger protein 214	mir-484
RNF215	ring finger protein 215	miR-26a
RNF24	ring finger protein 24	mir-484
RNF4	ring finger protein 4	miR-222
RNF44	ring finger protein 44	miR-222

RNF6	ring finger protein (C3H2C3 type) 6	miR-26a
RNFT2	ring finger protein, transmembrane 2	mir-484
RNPS1	RNA binding protein S1, serine-rich domain	miR-222
ROCK1	Rho-associated, coiled-coil containing protein kinase 1	miR-26a
RPGR	retinitis pigmentosa GTPase regulator	miR-26a
RPRD2	regulation of nuclear pre-mRNA domain containing 2	miR-26a
RPRM	reprimo, TP53 dependent G2 arrest mediator candi-date	mir-484
RPS6KA1	ribosomal protein S6 kinase, 90kDa, polypeptide 1	mir-484
RPS6KA2	ribosomal protein S6 kinase, 90kDa, polypeptide 2	miR-26a
RPS6KA6	ribosomal protein S6 kinase, 90kDa, polypeptide 6	miR-26a
RRAGD	Ras-related GTP binding D	mir-484
RREB1	ras responsive element binding protein 1	miR-222
RSBN1L	round spermatid basic protein 1-like	miR-222
RSPH9	radial spoke head 9 homolog (Chlamydomonas)	mir-484
RSPRY1	ring finger and SPRY domain containing 1	miR-26a
RTF1	Rtf1, Paf1/RNA polymerase II complex component, homolog (S. cerevisiae)	miR-26a
RTN1	reticulon 1	miR-26a
RUFY4	RUN and FYVE domain containing 4	mir-484
RUNX1T1	runt-related transcription factor 1; translocated to, 1 (cyclin D-related)	miR-222
RUNX2	runt-related transcription factor 2	miR-222
RWDD4	RWD domain containing 4	miR-26a
RYK	receptor-like tyrosine kinase	miR-26a
SACS	spastic ataxia of Charlevoix-Saguenay (sacsin)	miR-26a
SALL1	sal-like 1 (Drosophila)	miR-26a
SAMD12	sterile alpha motif domain containing 12	miR-26a
SAMD8	sterile alpha motif domain containing 8	miR-26a
SAP30L	SAP30-like	mir-484
SAR1B	SAR1 homolog B (S. cerevisiae)	miR-26a
SBK1	SH3 domain binding kinase 1	miR-222; miR-484
SBNO1	strawberry notch homolog 1 (Drosophila)	miR-26a
SCAI	suppressor of cancer cell invasion	miR-26a
SCARA3	scavenger receptor class A, member 3	mir-484
SCARB2	scavenger receptor class B, member 2	mir-484
SCD5	stearoyl-CoA desaturase 5	miR-222
SCML4	sex comb on midleg-like 4 (Drosophila)	miR-26a
SCN2B	sodium channel, voltage-gated, type II, beta subunit	mir-484
SCN5A	sodium channel, voltage-gated, type V, alpha subunit	miR-26a
SCOC	short coiled-coil protein	miR-26a
SCRIB	scribbled planar cell polarity protein	mir-484
SCRT2	scratch homolog 2, zinc finger protein (Drosophila)	mir-484
SCT	secretin	mir-484
SCUBE3	signal peptide, CUB domain, EGF-like 3	mir-484
SDC1	syndecan 1	mir-484

SEC16B	SEC16 homolog B (S. cerevisiae)	mir-484
SEC24A	SEC24 family, member A (S. cerevisiae)	miR-26a
SEC61A1	Sec61 alpha 1 subunit (S. cerevisiae)	mir-484
SEC62	SEC62 homolog (S. cerevisiae)	miR-222
SEMA4D	sema domain, immunoglobulin domain (Ig), transmembrane domain (TM) and short cytoplasmic domain, (semaphorin) 4D	mir-484
SEMA6D	sema domain, transmembrane domain (TM), and cytoplasmic domain, (semaphorin) 6D	miR-26a; miR-222
SENP5	SUMO1/sentrin specific peptidase 5	miR-26a
SENP6	SUMO1/sentrin specific peptidase 6	mir-484
SERBP1	SERPINE1 mRNA binding protein 1	miR-26a
SERP1	stress-associated endoplasmic reticulum protein 1	miR-26a
SERPINA3	serpin peptidase inhibitor, clade A (alpha-1 antiproteinase, antitrypsin), member 3	mir-484
SESN3	sestrin 3	miR-26a; miR-222
SESTD1	SEC14 and spectrin domains 1	miR-26a; miR-484
SETD8	SET domain containing (lysine methyltransferase) 8	miR-26a
SFMBT2	Scm-like with four mbt domains 2	miR-26a
SFRP5	secreted frizzled-related protein 5	mir-484
SFT2D2	SFT2 domain containing 2	mir-484
SFXN1	sideroflexin 1	mir-484
SH3BP4	SH3-domain binding protein 4	miR-222
SH3D19	SH3 domain containing 19	miR-26a
SH3PXD2A	SH3 and PX domains 2A	miR-26a
SH3RF1	SH3 domain containing ring finger 1	miR-26a
SH3RF3	SH3 domain containing ring finger 3	mir-484
SHANK2	SH3 and multiple ankyrin repeat domains 2	miR-222
SHC1	SHC (Src homology 2 domain containing) transforming protein 1	mir-484
SHC4	SHC (Src homology 2 domain containing) family, member 4	miR-26a
SHH	sonic hedgehog	mir-484
SHISA4	shisa family member 4	mir-484
SHISA5	shisa family member 5	mir-484
SHPK	transient receptor potential cation channel subfamily V member 1	mir-484
SIAH3	siah E3 ubiquitin protein ligase family member 3	mir-484
SIGMAR1	sigma non-opioid intracellular receptor 1	mir-484
SKAP2	src kinase associated phosphoprotein 2	mir-484
SKP2	S-phase kinase-associated protein 2, E3 ubiquitin protein ligase	miR-26a
SLC10A4	solute carrier family 10, member 4	miR-26a
SLC11A1	solute carrier family 11 (proton-coupled divalent metal ion transporter), member 1	mir-484

SLC12A2	solute carrier family 12 (sodium/potassium/chloride transporter), member 2	miR-26a
SLC16A6	solute carrier family 16, member 6	miR-26a
SLC17A9	solute carrier family 17 (vesicular nucleotide transporter), member 9	mir-484
SLC19A1	solute carrier family 19 (folate transporter), member 1	mir-484
SLC19A2	solute carrier family 19 (thiamine transporter), member 2	miR-26a
SLC1A1	solute carrier family 1 (neuronal/epithelial high affinity glutamate transporter, system Xag), member 1	miR-26a
SLC22A23	solute carrier family 22, member 23	miR-26a
SLC24A4	solute carrier family 24 (sodium/potassium/calcium exchanger), member 4	miR-26a
SLC25A16	solute carrier family 25 (mitochondrial carrier; Graves disease autoantigen), member 16	miR-26a
SLC25A19	solute carrier family 25 (mitochondrial thiamine pyrophosphate carrier), member 19	mir-484
SLC25A20	solute carrier family 25 (carnitine/acylcarnitine translocase), member 20	miR-26a
SLC25A37	solute carrier family 25 (mitochondrial iron transporter), member 37	miR-26a; miR-222
SLC26A4	solute carrier family 26 (anion exchanger), member 4	miR-26a
SLC2A13	solute carrier family 2 (facilitated glucose transporter), member 13	miR-26a
SLC2A5	solute carrier family 2 (facilitated glucose/fructose transporter), member 5	mir-484
SLC30A6	solute carrier family 30 (zinc transporter), member 6	mir-484
SLC34A2	solute carrier family 34 (type II sodium/phosphate contransporter), member 2	mir-484
SLC35B3	solute carrier family 35 (adenosine 3'-phospho 5'-phosphosulfate transporter), member B3	mir-484
SLC35C2	solute carrier family 35 (GDP-fucose transporter), member C2	mir-484
SLC35E2	solute carrier family 35, member E2	miR-26a
SLC35E2B	solute carrier family 35, member E2B	miR-26a
SLC35F2	solute carrier family 35, member F2	mir-484
SLC35G1	solute carrier family 35, member G1	miR-26a
SLC38A1	solute carrier family 38, member 1	miR-26a; miR-222; miR-484
SLC38A2	solute carrier family 38, member 2	miR-26a
SLC41A3	solute carrier family 41, member 3	mir-484
SLC45A4	solute carrier family 45, member 4	miR-26a; miR-484
SLC46A1	solute carrier family 46 (folate transporter), member 1	mir-484
SLC4A4	solute carrier family 4 (sodium bicarbonate cotransporter), member 4	miR-26a; miR-222
SLC5A10	solute carrier family 5 (sodium/sugar cotransporter), member 10	mir-484

SLC6A1	solute carrier family 6 (neurotransmitter transporter), member 1	miR-222
SLC6A15	solute carrier family 6 (neutral amino acid transporter), member 15	miR-26a
SLC6A6	solute carrier family 6 (neurotransmitter transporter), member 6	miR-26a
SLC7A11	solute carrier family 7 (anionic amino acid transporter light chain, xc- system), member 11	miR-26a
SLC9A2	solute carrier family 9, subfamily A (NHE2, cation proton antiporter 2), member 2	miR-26a
SLCO1A2	solute carrier organic anion transporter family, member 1A2	miR-222
SLCO5A1	solute carrier organic anion transporter family, member 5A1	miR-26a
SLFN12	schlafen family member 12	mir-484
SLITRK5	SLIT and NTRK-like family, member 5	miR-222
SLK	STE20-like kinase	mir-484
SMAD1	SMAD family member 1	miR-26a
SMARCA5	SWI/SNF related, matrix associated, actin dependent regulator of chromatin, subfamily a, member 5	miR-222
SMARCE1	SWI/SNF related, matrix associated, actin dependent regulator of chromatin, subfamily e, member 1	mir-484
SMG6	SMG6 nonsense mediated mRNA decay factor	mir-484
SMIM12	small integral membrane protein 12	mir-484
SMNDC1	survival motor neuron domain containing 1	miR-26a; miR-222
SNAP29	synaptosomal-associated protein, 29kDa	miR-222
SNCB	synuclein, beta	miR-222
SNN	stannin	miR-26a
SNX2	sorting nexin 2	mir-484
SNX21	sorting nexin family member 21	mir-484
SNX30	sorting nexin family member 30	miR-26a
SNX33	sorting nexin 33	mir-484
SOCS1	suppressor of cytokine signaling 1	miR-222
SOCS5	suppressor of cytokine signaling 5	miR-26a
SOCS6	suppressor of cytokine signaling 6	miR-26a
SOCS7	suppressor of cytokine signaling 7	miR-26a; miR-222
SOGA3	SOGA family member 3	miR-26a
SOSTDC1	sclerostin domain containing 1	miR-26a
SOX1	SRY (sex determining region Y)-box 1	miR-222
SOX17	SRY (sex determining region Y)-box 17	miR-26a
SOX5	SRY (sex determining region Y)-box 5	miR-26a
SPATS2	spermatogenesis associated, serine-rich 2	mir-484
SPATS2L	spermatogenesis associated, serine-rich 2-like	miR-222
SPIN1	spindlin 1	mir-484
SPOCD1	SPOC domain containing 1	mir-484

SPOCK2	sparc/osteonectin, cwcv and kazal-like domains proteoglycan (testican) 2	miR-26a
SPPL3	signal peptide peptidase like 3	miR-222
SPRED1	sprouty-related, EVH1 domain containing 1	miR-222
SPRED2	sprouty-related, EVH1 domain containing 2	miR-222
SPTSSA	serine palmitoyltransferase, small subunit A	miR-222
SRCAP	Snf2-related CREBBP activator protein	miR-26a
SRGAP1	SLIT-ROBO Rho GTPase activating protein 1	miR-26a; miR-484
SRGAP2	SLIT-ROBO Rho GTPase activating protein 2	mir-484
SRP19	signal recognition particle 19kDa	miR-26a
SRSF10	serine/arginine-rich splicing factor 10	miR-26a
SRSF2	serine/arginine-rich splicing factor 2	miR-222
SRSF4	serine/arginine-rich splicing factor 4	mir-484
SRSF7	serine/arginine-rich splicing factor 7	miR-222
SSFA2	sperm specific antigen 2	miR-26a
SSX2IP	synovial sarcoma, X breakpoint 2 interacting protein	miR-26a
ST3GAL6	ST3 beta-galactoside alpha-2,3-sialyltransferase 6	miR-26a
ST6GAL2	ST6 beta-galactosamide alpha-2,6-sialyltranferase 2	miR-26a
ST8SIA1	ST8 alpha-N-acetyl-neuraminide alpha-2,8-sialyltransferase 1	miR-222; miR-484
ST8SIA4	ST8 alpha-N-acetyl-neuraminide alpha-2,8-sialyltransferase 4	miR-26a
STAC2	SH3 and cysteine rich domain 2	miR-26a
STARD13	StAR-related lipid transfer (START) domain containing 13	mir-484
STEAP2	STEAP family member 2, metalloreductase	mir-484
STEAP3	STEAP family member 3, metalloreductase	mir-484
STK11	serine/threonine kinase 11	miR-26a
STK39	serine threonine kinase 39	miR-26a
STMN1	stathmin 1	miR-26a
STOML1	stomatin (EPB72)-like 1	mir-484
STOX2	storkhead box 2	miR-26a
STRA6	stimulated by retinoic acid 6	mir-484
STRADB	STE20-related kinase adaptor beta	miR-26a
STRBP	spermatid perinuclear RNA binding protein	miR-26a
STT3B	STT3B, subunit of the oligosaccharyltransferase complex (catalytic)	miR-26a
STX17	syntaxin 17	mir-484
STX1B	syntaxin 1B	miR-222
STX7	syntaxin 7	miR-26a
STYX	serine/threonine/tyrosine interacting protein	miR-26a; miR-222; miR-484
SULF1	sulfatase 1	miR-26a
SULT4A1	sulfotransferase family 4A, member 1	mir-484
SUN2	Sad1 and UNC84 domain containing 2	miR-222

SUSD1	sushi domain containing 1	mir-484
SUV420H2	suppressor of variegation 4-20 homolog 2 (Drosophila)	miR-26a
SYNCRIP	synaptotagmin binding, cytoplasmic RNA interacting protein	miR-26a
SYNPO	synaptopodin	mir-484
SYT1	synaptotagmin I	mir-484
SYT10	synaptotagmin X	miR-26a
SYT15	synaptotagmin XV	mir-484
TAB2	TGF-beta activated kinase 1/MAP3K7 binding protein 2	miR-26a
TAB3	TGF-beta activated kinase 1/MAP3K7 binding protein 3	miR-26a
TAF9B	TAF9B RNA polymerase II, TATA box binding protein (TBP)-associated factor, 31kDa	miR-26a
TANC2	tetratricopeptide repeat, ankyrin repeat and coiled-coil containing 2	miR-26a
TAOK1	TAO kinase 1	miR-26a; miR-222
TARBP2	TAR (HIV-1) RNA binding protein 2	mir-484
TBC1D25	TBC1 domain family, member 25	mir-484
TBC1D30	TBC1 domain family, member 30	miR-26a
TBC1D4	TBC1 domain family, member 4	miR-26a
TBX15	T-box 15	miR-222
TCEA1	transcription elongation factor A (SII), 1	mir-484
TCEANC2	transcription elongation factor A (SII) N-terminal and central domain containing 2	mir-484
TCF12	transcription factor 12	miR-26a; miR-222
TCF21	transcription factor 21	mir-484
TCF4	transcription factor 4	miR-26a; miR-222
TCF7	transcription factor 7 (T-cell specific, HMG-box)	mir-484
TCF7L2	transcription factor 7-like 2 (T-cell specific, HMG-box)	miR-26a; miR-222
TCIRG1	T-cell, immune regulator 1, ATPase, H+ transporting, lysosomal V0 subunit A3	mir-484
TDRD10	tudor domain containing 10	mir-484
TECPR1	tectonin beta-propeller repeat containing 1	mir-484
TERF2	telomeric repeat binding factor 2	miR-222
TESK2	testis-specific kinase 2	miR-26a
TET1	tet methylcytosine dioxygenase 1	miR-26a
TET2	tet methylcytosine dioxygenase 2	miR-26a
TET3	tet methylcytosine dioxygenase 3	miR-26a
TEX261	testis expressed 261	mir-484
TFAP2A	transcription factor AP-2 alpha (activating enhancer binding protein 2 alpha)	miR-26a
TFAP2C	transcription factor AP-2 gamma (activating enhancer binding protein 2 gamma)	miR-26a

TFAP2E	transcription factor AP-2 epsilon (activating enhancer binding protein 2 epsilon)	miR-26a
TGFA	transforming growth factor, alpha	mir-484
TGFB1	transforming growth factor, beta 1	mir-484
TGFB2	transforming growth factor, beta 2	mir-484
TGIF2-C20orf24	TGIF2-C20orf24 readthrough	miR-26a
THAP2	THAP domain containing, apoptosis associated protein 2	miR-26a
THAP4	THAP domain containing 4	mir-484
THBD	thrombomodulin	mir-484
THRAP3	thyroid hormone receptor associated protein 3	miR-26a
TIFAB	TRAF-interacting protein with forkhead-associated domain, family member B	mir-484
TIMP2	TIMP metallopeptidase inhibitor 2	mir-484
TIMP3	TIMP metallopeptidase inhibitor 3	miR-222
TIPARP	TCDD-inducible poly(ADP-ribose) polymerase	miR-222
TIRAP	toll-interleukin 1 receptor (TIR) domain containing adaptor protein	mir-484
TLE3	transducin-like enhancer of split 3 (E(sp1) homolog, Drosophila)	miR-222
TLK1	tousled-like kinase 1	miR-222
TLR3	toll-like receptor 3	miR-26a
TLX1	T-cell leukemia homeobox 1	mir-484
TM7SF2	transmembrane 7 superfamily member 2	mir-484
TMC7	transmembrane channel-like 7	miR-26a
TMCC1	transmembrane and coiled-coil domain family 1	miR-26a; miR-222
TMCO4	transmembrane and coiled-coil domains 4	mir-484
TMED10	transmembrane emp24-like trafficking protein 10 (yeast)	miR-26a
TMEM117	transmembrane protein 117	mir-484
TMEM135	transmembrane protein 135	miR-26a
TMEM178B	transmembrane protein 178B	miR-26a
TMEM180	transmembrane protein 180	mir-484
TMEM184B	transmembrane protein 184B	miR-26a
TMEM189	transmembrane protein 189	mir-484
TMEM2	transmembrane protein 2	miR-26a
TMEM200B	transmembrane protein 200B	miR-26a
TMEM208	transmembrane protein 208	mir-484
TMEM239	transmembrane protein 239	mir-484
TMEM242	transmembrane protein 242	mir-484
TMEM251	transmembrane protein 251	miR-26a
TMEM260	transmembrane protein 260	miR-26a
TMEM33	transmembrane protein 33	miR-26a
TMEM43	transmembrane protein 43	mir-484
TMEM52	transmembrane protein 52	mir-484
TMEM56	transmembrane protein 56	miR-26a

TMEM63C	transmembrane protein 63C	mir-484
TMEM64	transmembrane protein 64	miR-26a
TMEM68	transmembrane protein 68	miR-26a
TMOD3	tropomodulin 3 (ubiquitous)	mir-484
TMOD4	tropomodulin 4 (muscle)	mir-484
TMTC1	transmembrane and tetratricopeptide repeat containing 1	mir-484
TMTC3	transmembrane and tetratricopeptide repeat containing 3	miR-26a
TNFAIP8	tumor necrosis factor, alpha-induced protein 8	mir-484
TNKS2	tankyrase, TRF1-interacting ankyrin-related ADP-ribose polymerase 2	miR-26a
TNPO1	transportin 1	miR-26a
TNRC6A	trinucleotide repeat containing 6A	miR-26a
TNRC6B	trinucleotide repeat containing 6B	miR-26a
TNRC6C	trinucleotide repeat containing 6C	miR-26a; miR-222
TOB1	transducer of ERBB2, 1	miR-26a
TOR1AIP1	torsin A interacting protein 1	miR-222
TOX	thymocyte selection-associated high mobility group box	miR-222
TP53AIP1	tumor protein p53 regulated apoptosis inducing protein 1	mir-484
TP53BP2	tumor protein p53 binding protein, 2	miR-222
TP53INP1	tumor protein p53 inducible nuclear protein 1	miR-26a
TP53INP2	tumor protein p53 inducible nuclear protein 2	miR-26a; miR-222; miR-484
TPD52	tumor protein D52	miR-26a
TPP2	tripeptidyl peptidase II	mir-484
TPPP	tubulin polymerization promoting protein	miR-26a
TRABD2B	TraB domain containing 2B	miR-222
TRAPPC2L	trafficking protein particle complex 2-like	mir-484
TRAPPC3	trafficking protein particle complex 3	mir-484
TRERF1	transcriptional regulating factor 1	miR-26a
TRIB1	tribbles pseudokinase 1	mir-484
TRIB2	tribbles pseudokinase 2	miR-26a; miR-222
TRIM46	tripartite motif containing 46	mir-484
TRIM71	tripartite motif containing 71, E3 ubiquitin protein ligase	miR-26a
TRPC3	transient receptor potential cation channel, subfamily C, member 3	miR-26a; miR-222
TRPC4AP	transient receptor potential cation channel, subfamily C, member 4 associated protein	mir-484
TRPC6	transient receptor potential cation channel, subfamily C, member 6	miR-26a
TRPM3	transient receptor potential cation channel, subfamily M, member 3	miR-222

TRPM6	transient receptor potential cation channel, subfamily M, member 6	miR-26a
TRPS1	trichorhinophalangeal syndrome I	miR-26a; miR-222
TRPV1	transient receptor potential cation channel, subfamily V, member 1	mir-484
TSC22D2	TSC22 domain family, member 2	miR-26a
TSC22D3	TSC22 domain family, member 3	miR-222
TSC22D4	TSC22 domain family, member 4	miR-26a
TSNARE1	t-SNARE domain containing 1	mir-484
TSPAN14	tetraspanin 14	miR-26a
TSPAN3	tetraspanin 3	mir-484
TSPYL4	TSPY-like 4	miR-26a; miR-484
TTC13	tetratricopeptide repeat domain 13	miR-26a
TTC28	tetratricopeptide repeat domain 28	miR-26a
TTC9C	tetratricopeptide repeat domain 9C	miR-26a
TTPAL	tocopherol (alpha) transfer protein-like	miR-26a
TUB	tubby bipartite transcription factor	miR-26a
TUBD1	tubulin, delta 1	miR-26a
TUFT1	tuftelin 1	mir-484
TWF1	twinfilin actin-binding protein 1	miR-26a; miR-484
TXNDC15	thioredoxin domain containing 15	miR-26a
UBAC1	UBA domain containing 1	miR-26a
UBAP2L	ubiquitin associated protein 2-like	mir-484
UBE2D1	ubiquitin-conjugating enzyme E2D 1	miR-26a
UBE2D4	ubiquitin-conjugating enzyme E2D 4 (putative)	mir-484
UBE2E2	ubiquitin-conjugating enzyme E2E 2	miR-26a
UBE2G1	ubiquitin-conjugating enzyme E2G 1	miR-26a; miR-222
UBE2H	ubiquitin-conjugating enzyme E2H	miR-26a
UBE2J1	ubiquitin-conjugating enzyme E2, J1	miR-26a; miR-222
UBE2QL1	ubiquitin-conjugating enzyme E2Q family-like 1	miR-26a
UBE2R2	ubiquitin-conjugating enzyme E2R 2	mir-484
UBE2V1	ubiquitin-conjugating enzyme E2 variant 1	mir-484
UBE2W	ubiquitin-conjugating enzyme E2W (putative)	miR-26a
UBE3A	ubiquitin protein ligase E3A	miR-26a
UBE4B	ubiquitination factor E4B	miR-26a
UBN2	ubinuclein 2	miR-26a; miR-222; miR-484
UBQLN4	ubiquilin 4	mir-484
UBR3	ubiquitin protein ligase E3 component n-recognin 3 (putative)	miR-26a
UBTD2	ubiquitin domain containing 2	miR-26a
UBXN7	UBX domain protein 7	mir-484

UCK2	uridine-cytidine kinase 2	miR-26a
UGT8	UDP glycosyltransferase 8	miR-26a
ULK1	unc-51 like autophagy activating kinase 1	miR-26a
ULK2	unc-51 like autophagy activating kinase 2	miR-26a
UNC45A	unc-45 homolog A (C. elegans)	mir-484
URI1	URI1, prefoldin-like chaperone	miR-222
USP15	ubiquitin specific peptidase 15	miR-26a
USP2	ubiquitin specific peptidase 2	mir-484
USP24	ubiquitin specific peptidase 24	mir-484
USP25	ubiquitin specific peptidase 25	miR-26a
USP27X	ubiquitin specific peptidase 27, X-linked	miR-26a
USP3	ubiquitin specific peptidase 3	miR-26a
USP37	ubiquitin specific peptidase 37	miR-26a
USP53	ubiquitin specific peptidase 53	miR-26a
USP9X	ubiquitin specific peptidase 9, X-linked	miR-26a
USP9Y	ubiquitin specific peptidase 9, Y-linked	miR-26a
UST	uronyl-2-sulfotransferase	mir-484
UTY	ubiquitously transcribed tetratricopeptide repeat containing, Y-linked	miR-26a
VAC14	Vac14 homolog (S. cerevisiae)	mir-484
VAMP1	vesicle-associated membrane protein 1 (synaptobrevin 1)	miR-26a
VAMP2	vesicle-associated membrane protein 2 (synaptobrevin 2)	mir-484
VANGL2	VANGL planar cell polarity protein 2	miR-26a
VAPB	VAMP (vesicle-associated membrane protein)-associated protein B and C	miR-222
VASH1	vasohibin 1	miR-222
VASH2	vasohibin 2	mir-484
VAV3	vav 3 guanine nucleotide exchange factor	mir-484
VDAC1	voltage-dependent anion channel 1	miR-26a
VEZF1	vascular endothelial zinc finger 1	miR-222
VGLL4	vestigial like 4 (Drosophila)	miR-222
VLDLR	very low density lipoprotein receptor	miR-26a
VMP1	vacuole membrane protein 1	miR-26a
VPS37D	vacuolar protein sorting 37 homolog D (S. cerevisiae)	mir-484
VSIG10	V-set and immunoglobulin domain containing 10	miR-26a
VTI1A	vesicle transport through interaction with t-SNAREs 1A	mir-484
VTI1B	vesicle transport through interaction with t-SNAREs 1B	miR-26a
WAPAL	wings apart-like homolog (Drosophila)	miR-26a
WASF2	WAS protein family, member 2	miR-222
WDFY2	WD repeat and FYVE domain containing 2	mir-484
WDFY4	WDFY family member 4	mir-484
WDR31	WD repeat domain 31	mir-484
WDR47	WD repeat domain 47	miR-222

WDR6	WD repeat domain 6	mir-484
WDR72	WD repeat domain 72	mir-484
WHSC1	Wolf-Hirschhorn syndrome candidate 1	miR-26a
WIPF2	WAS/WASL interacting protein family, member 2	miR-26a
WLS	wntless Wnt ligand secretion mediator	miR-222
WNK1	WNK lysine deficient protein kinase 1	miR-26a
WNK3	WNK lysine deficient protein kinase 3	miR-26a; miR-222
WNT4	wingless-type MMTV integration site family, member 4	mir-484
WNT5A	wingless-type MMTV integration site family, member 5A	miR-26a
WNT9A	wingless-type MMTV integration site family, member 9A	mir-484
WNT9B	wingless-type MMTV integration site family, member 9B	mir-484
XKR4	XK, Kell blood group complex subunit-related family, member 4	miR-26a; miR-222
XPO7	exportin 7	mir-484
YAP1	Yes-associated protein 1	mir-484
YDJC	YdjC homolog (bacterial)	mir-484
YIPF4	Yip1 domain family, member 4	miR-26a
YPEL1	yippee-like 1 (Drosophila)	miR-26a
YTHDC1	YTH domain containing 1	miR-222
YTHDF3	YTH domain family, member 3	miR-26a
YWHAE	tyrosine 3-monooxygenase/tryptophan 5-monooxygenase activation protein, epsilon polypeptide	miR-26a
YWHAG	tyrosine 3-monooxygenase/tryptophan 5-monooxygenase activation protein, gamma polypeptide	miR-222
ZADH2	zinc binding alcohol dehydrogenase domain containing 2	miR-222
ZBTB10	zinc finger and BTB domain containing 10	miR-26a
ZBTB16	zinc finger and BTB domain containing 16	miR-26a
ZBTB18	zinc finger and BTB domain containing 18	miR-26a
ZBTB20	zinc finger and BTB domain containing 20	miR-26a
ZBTB37	zinc finger and BTB domain containing 37	miR-26a; miR-222
ZBTB38	zinc finger and BTB domain containing 38	miR-26a; miR-484
ZBTB42	zinc finger and BTB domain containing 42	mir-484
ZBTB7C	zinc finger and BTB domain containing 7C	miR-26a
ZC3H7B	zinc finger CCCH-type containing 7B	miR-26a
ZCCHC11	zinc finger, CCHC domain containing 11	miR-26a
ZCCHC14	zinc finger, CCHC domain containing 14	mir-484
ZCCHC17	zinc finger, CCHC domain containing 17	mir-484
ZCCHC2	zinc finger, CCHC domain containing 2	miR-26a
ZCCHC24	zinc finger, CCHC domain containing 24	miR-26a
ZDHHC17	zinc finger, DHHC-type containing 17	miR-222

ZDHHC18	zinc finger, DHHC-type containing 18	miR-26a; miR-484
ZDHHC20	zinc finger, DHHC-type containing 20	miR-26a
ZDHHC23	zinc finger, DHHC-type containing 23	mir-484
ZDHHC3	zinc finger, DHHC-type containing 3	mir-484
ZDHHC6	zinc finger, DHHC-type containing 6	miR-26a
ZDHHC7	zinc finger, DHHC-type containing 7	miR-26a
ZDHHC8	zinc finger, DHHC-type containing 8	mir-484
ZDHHC9	zinc finger, DHHC-type containing 9	mir-484
ZEB2	zinc finger E-box binding homeobox 2	miR-26a; miR-222
ZFAND2B	zinc finger, AN1-type domain 2B	mir-484
ZFAND5	zinc finger, AN1-type domain 5	miR-222
ZFC3H1	zinc finger, C3H1-type containing	miR-26a
ZFHX2	zinc finger homeobox 2	mir-484
ZFHX3	zinc finger homeobox 3	miR-26a; miR-222
ZFHX4	zinc finger homeobox 4	miR-26a
ZFP36	ZFP36 ring finger protein	miR-222
ZFP36L2	ZFP36 ring finger protein-like 2	miR-222
ZFPM2	zinc finger protein, FOG family member 2	miR-222
ZFX	zinc finger protein, X-linked	miR-26a
ZFY	zinc finger protein, Y-linked	miR-26a
ZFYVE1	zinc finger, FYVE domain containing 1	mir-484
ZIC4	Zic family member 4	mir-484
ZIC5	Zic family member 5	miR-26a
ZKSCAN1	zinc finger with KRAB and SCAN domains 1	miR-26a
ZMAT5	zinc finger, matrin-type 5	mir-484
ZMIZ2	zinc finger, MIZ-type containing 2	mir-484
ZMYM2	zinc finger, MYM-type 2	miR-222
ZNF148	zinc finger protein 148	miR-26a
ZNF217	zinc finger protein 217	miR-26a
ZNF275	zinc finger protein 275	miR-26a
ZNF385A	zinc finger protein 385A	miR-222
ZNF385B	zinc finger protein 385B	miR-26a
ZNF410	zinc finger protein 410	miR-26a
ZNF462	zinc finger protein 462	miR-26a
ZNF469	zinc finger protein 469	miR-26a
ZNF503	zinc finger protein 503	mir-484
ZNF516	zinc finger protein 516	miR-26a
ZNF518B	zinc finger protein 518B	miR-222
ZNF574	zinc finger protein 574	mir-484
ZNF598	zinc finger protein 598	miR-26a
ZNF608	zinc finger protein 608	miR-26a
ZNF618	zinc finger protein 618	miR-222
ZNF629	zinc finger protein 629	miR-222; miR-484

ZNF641	zinc finger protein 641	mir-484
ZNF652	zinc finger protein 652	miR-26a; miR-222
ZNF697	zinc finger protein 697	miR-26a
ZNF783	zinc finger family member 783	mir-484
ZNF784	zinc finger protein 784	mir-484
ZNF786	zinc finger protein 786	mir-484
ZNRF3	zinc and ring finger 3	miR-26a
ZSWIM6	zinc finger, SWIM-type containing 6	miR-26a
